# Community engagement interventions for communicable disease control in low- and lower- middle-income countries: evidence from a review of systematic reviews

**DOI:** 10.1186/s12939-020-01169-5

**Published:** 2020-04-06

**Authors:** K. Questa, M. Das, R. King, M. Everitt, C. Rassi, C. Cartwright, T. Ferdous, D. Barua, N. Putnis, A. C. Snell, R. Huque, J. Newell, H. Elsey

**Affiliations:** 1 Nuffield Centre for International Health and Development, University of Leeds, Room 1029, Level 10, Worsley Building, Leeds, LS2 9NL UK; 2 Malaria Consortium, London, UK; 3 ARK Foundation, Dhaka, Bangladesh; 4 University of York, York, UK

**Keywords:** Community engagement, Communicable diseases, Low and lower-middle-income countries, Umbrella review

## Abstract

**Background:**

Community engagement (CE) interventions include a range of approaches to involve communities in the improvement of their health and wellbeing. Working with communities defined by location or some other shared interest, these interventions may be important in assisting equity and reach of communicable disease control (CDC) in low and lower-middle income countries (LLMIC). We conducted an umbrella review to identify approaches to CE in communicable disease control, effectiveness of these approaches, mechanisms and factors influencing success.

**Methods:**

We included systematic reviews that: i) focussed on CE interventions; ii) involved adult community members; iii) included outcomes relevant to communicable diseases in LLMIC; iv) were written in English. Quantitative results were extracted and synthesised narratively. A qualitative synthesis process enabled identification of mechanisms of effect and influencing factors. We followed guidance from the Joanna Briggs Institute, assessed quality with the DARE tool and reported according to standard systematic review methodology.

**Results:**

Thirteen systematic reviews of medium-to-high quality were identified between June and July 2017. Reviews covered the following outcomes: HIV and STIs (6); malaria (2); TB (1); child and maternal health (3) and mixed (1). Approaches included: CE through peer education and community health workers, community empowerment interventions and more general community participation or mobilisation. Techniques included sensitisation with the community and involvement in the identification of resources, intervention development and delivery. Evidence of effectiveness of CE on health outcomes was mixed and quality of primary studies variable. We found: i) significantly reduced neonatal mortality following women’s participatory learning and action groups; ii) significant reductions in HIV and other STIs with empowerment and mobilisation interventions with marginalised groups; iii) significant reductions in malaria incidence or prevalence in a small number of primary studies; iv) significant reductions in infant diarrhoea following community health worker interventions. Mechanisms of impact commonly occurred through social and behavioural processes, particularly: changing social norms, increasing social cohesion and social capacity. Factors influencing effectiveness of CE interventions included extent of population coverage, shared leadership and community control over outcomes.

**Conclusion:**

Community engagement interventions may be effective in supporting CDC in LLMIC. Careful design of CE interventions appropriate to context, disease and community is vital.

## Background

Infectious diseases remain a major contributor to death and disability across the globe, with a greater proportion of disease and economic burden occurring in low and lower-middle-income countries (LLMIC). Progress has been made in the detection, treatment and prevention of key communicable diseases such as HIV, malaria and TB [1]. However further work is required to meet the 2015–2030 Sustainable Development Goals (SDGs). SDG 3 focuses global attention on infectious diseases with the target (3.3) of ending the epidemics of AIDS, tuberculosis, malaria and neglected tropical diseases by 2030 and combating hepatitis, water-borne diseases and other communicable diseases [1]. The further commitment of UN member states to ensure universal health coverage to all their citizens has focussed attention on approaches such as community engagement. Understanding when, how and for whom, community engagement can be effective in responding to infectious diseases is vital to inform efforts to meet the SDGs and understanding the limitations in the current evidence base is needed to inform the focus of future research.

In low and lower-middle-income countries, community engagement (CE) initiatives have been described as ‘critical enablers’ in the response to communicable diseases (CDs) [2–5]. Such initiatives may be particularly important in settings where health systems are under-resourced, and the collective capacity of communities becomes a key resource in effecting behaviour change and delivering health outcomes [6, 7]. With regard to health equity, there is also some evidence to suggest CE may be effective in the prevention and management of communicable disease control (CDC) in marginalised groups [8, 9]. However, CE is a broad topic, with many different delivery mechanisms and techniques. For example, ‘community participation’, community mobilisation’ and ‘community empowerment’ may all be classed under the wider umbrella term of community engagement [10]. A recent systematic review by O’Mara Eves et al. [10] presented a comprehensive overview of the effectiveness of community engagement interventions in OECD countries, but an equivalent overview of research is lacking in low and lower-middle-income countries [10].

We conducted an umbrella review of community engagement interventions for communicable disease control in low and lower-middle-income countries. Umbrella reviews follow a systematic review methodology to identify, quality assess and synthesise the results from existing reviews of literature [11]. Umbrella reviews have been increasingly used in public health research, proving particularly useful where existing research synthesis may vary in several dimensions [11]. We chose this methodology to enable an overview of two large topics: Communicable diseases and community engagement interventions.

Our key research questions were:

- Which community engagement approaches and techniques are used in communicable disease control in low and lower-middle-income countries, and what is the effectiveness of these approaches?
- What are the [proposed] mechanisms by which community engagement interventions lead to improvements in communicable disease control and management?
- Which population and contextual factors influence the effectiveness of community engagement interventions for communicable disease control?

Our research questions were intended to generate an overview of the existing evidence base of community engagement interventions for communicable disease control and to provide those planning community engagement interventions with an understanding of how the initiatives may work, to enable design and evaluation of such interventions.

## Methods

The review was registered with the Prospero database (CRD42017074134) and followed recommended guidance adapted for public health interventions [11, 12].

Inclusion criteria were systematic review papers [13] that i) focussed on CE interventions AND ii) involved adult members of the community, AND iii) included outcomes relevant to communicable disease control and management in LLMICs.

The CE interventions could be stand-alone or part of multi-component interventions and could be intended for child or adult health outcomes, as long as the participants themselves were adults.

To screen studies for eligibility, we used two definitions of CE, which both had to be met: a) ‘An umbrella term encompassing a continuum of approaches to engaging communities of place and/or interest in activities aimed at improving population health and/or reducing health inequalities’ [14] and b) ‘the process of working collaboratively with and through groups of people affiliated by geographic proximity, special interest, or similar situations to address issues affecting the well-being of those people’ [15]. This ensured a broad range of CE interventions would be captured, whilst distinguishing the studies from non-participatory approaches such as healthcare professional-led education.

Reviews were considered to ‘focus’ on community engagement if: i) review inclusion criteria provided a description of CE interventions in keeping with the above definitions [14, 15] and ii) all primary studies within the review had at least one component of community engagement.

We considered reviews of any type of study design, with any comparison group. No single definition of ‘adult’ participant was chosen – this was dependent on the definition used within each review. We used the World Bank (2017) definition of LLMICs, and the World Health Organisation (WHO) definition of communicable diseases [16].

We included reviews with the following direct or intermediate outcomes for communicable disease control: measures of communicable disease incidence, prevalence, morbidity or mortality, treatment uptake or adherence, or behaviours that could be clearly linked to communicable disease control (e.g handwashing, vaccination, condom use) [16].

We excluded reviews that solely reported outcomes with indirect relevance to communicable disease control (such as nutrition and breastfeeding) as being insufficiently specific to communicable disease control. We included reviews published from 2007 onwards as we considered this adequate to capture the recommended 30 years of primary research [11]. We only included reviews in English due to the nature of the research team and resources available.

We extracted data from the reviews that identified key mechanisms and theories underpinning CE interventions, as well as factors influencing their success or failure [12], since these are needed to support intervention development. To structure our data extraction and synthesis of findings we used the MRC process evaluation model structure and definitions of intervention and implementation, mechanisms, outcomes and context [17].

We included peer-reviewed and non-peer-reviewed studies. Between June and July 2017, we searched the following databases in Epub Ahead of Print, In-Process & Other Non-Indexed Citations: MEDLINE (R) Daily and MEDLINE (R) Embase Classic, Embase and Global Health Cochrane (Wiley) and Campbell Libraries and the 3ie website. We checked Google Scholar search engine and websites from key organisations (UNAIDS, WHO, UNDP, World Bank) for relevant non-peer reviewed studies.

We developed searches for the concepts LLMICs, CE strategies and communicable diseases (see supplementary material 1). Identified studies were initially screened by two authors on title and abstract for relevance against inclusion criteria. Two authors full-text screened those meeting the criteria, or lacking information, and discussed and resolved disagreements. Four authors piloted data extraction forms, and nine authors took part in data extraction with two researchers independently reviewing each paper. We extracted data on review aims, methods, number of included studies, number of included studies from LLMICs, study designs, definition of CE approaches used, underlying theoretical framework, context, mechanisms, study conclusions and limitations. For reviews synthesising quantitative results, we extracted outcomes measured and results. For qualitative and mixed methods reviews, we extracted qualitative themes. In cases where reviews presented a range of results from studies in different income settings, with communicable and non-communicable diseases outcomes, and both adult and child participants in interventions, we extracted only results relevant to our review. A filtering process was therefore applied to identify relevant primary studies within each systematic review that matched our overall inclusion criteria. Where quantitative results had been pooled in a meta-analysis, we extracted the pooled results only where all contributing primary studies met our inclusion criteria. We assessed the quality of each review using the adapted DARE tool [18] which has been previously used in an umbrella review of public health interventions [19]. We rated review quality as low (0–3), medium (4–5) or high (6–7). The quality of the reviews was considered when discussing the evidence generated.

To present quantitative and qualitative results from the review papers, we used a narrative synthesis approach, because of the diversity of review styles and outcomes. Where findings from primary research reached statistical significance, these were also summarised narratively in order to provide an overview of an extensive evidence base as concisely as possible. Details of the type of study design relating to each single result were not reported within the text of the paper, however, these details were reported in supporting tables to aid interpretation of findings.

In addition, to generate emergent qualitative themes that met our relevance criteria, we used a qualitative synthesis process guided by our adapted MRC model categories. Qualitative extracts were separated into single statements or topics. A team of four researchers then independently arranged the statements into possible themes under each of the model categories of: i) intervention, ii) mechanism iii) influencing factors and iv) proximal (or intermediary) outcomes. Themes under each category were emergent, based on similarities in extracts of texts. The extracts placed under each category were then compared and any disagreements resolved until all extracts were included within themes under each of the model categories. During this process the team identified a further category not explicit in the MRC model, of factors affecting sustainability and scalability of interventions. This category was felt to be distinct and as sustainability is clearly such an important factor, this was added to the MRC model.

## Results

### Study selection

**Fig. 1:**
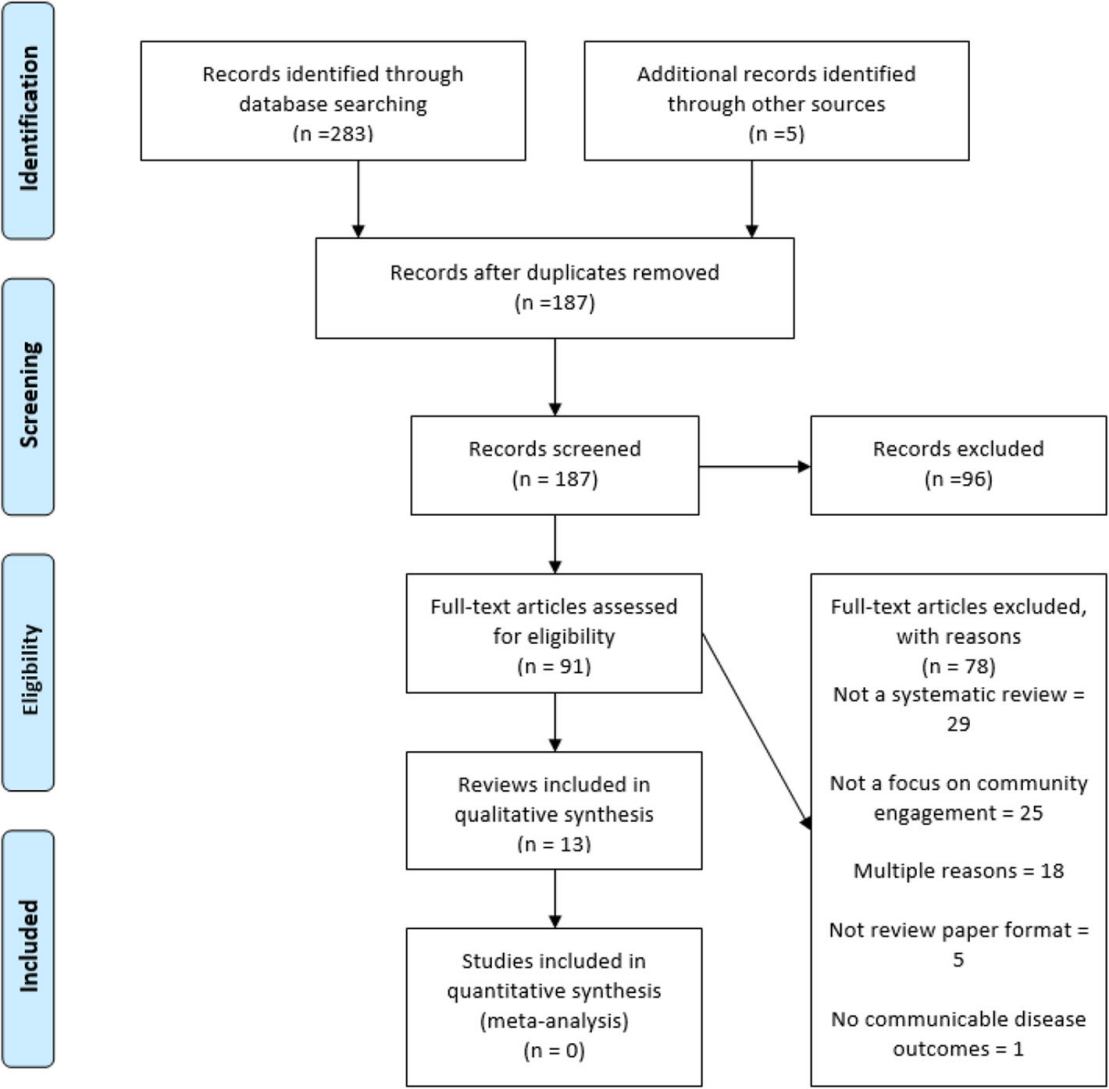
Flow diagram of systematic review selection

After removal of duplicates, 187 individual papers were screened. Ninety-six of these were excluded after review of title and abstract – only one of these was excluded on the basis of language alone. Full texts were examined for the remaining 91 papers. Thirteen reviews were included in the review (see Fig. 1).

### Characteristics of included reviews

The primary studies were undertaken within 50 different LLMICs, with India being the most common setting (*n* = 9) followed by Tanzania (*n* = 8) and Uganda (*n* = 7). Six of the studies focused on HIV [7–9, 20–22], three on child and maternal health [2, 23, 24] two on malaria [25, 26], one on TB [27] and one focused on birth related infection control practices and sexually transmitted infections (STIs) [28]. Target populations varied - some HIV reviews focused on high risk populations such as female sex workers (FSW) [8, 9], others looked at the general population including high risk groups [7, 20, 22] while one review focussed on people living with HIV and AIDS [21]. Reviews of malaria interventions studied general population groups [25, 26] and the single review of TB focussed on people living with TB [27]. The child and maternal health reviews typically included women of reproductive age, pregnant women as well as other members of the community [2, 23, 24].

The majority of review papers synthesised quantitative data only, with a few including a small number of qualitative or mixed methods primary studies. One review included only randomized controlled trials (RCTs) [23], the other reviews featured a variety of study designs with a relative lack of RCTs. Please see supplementary material 2 for a summary of the characteristics of the included reviews ().

### Review quality

Overall, quality of the included reviews was moderate to high with three of the reviews assessed as meeting all the quality criteria of the adapted DARE tool [8, 22, 23]. Table 1 provides a breakdown of the scores for each review. However, quality of the primary studies within the reviews was more varied and often poor where it was reported. Only one review presented evidence entirely from RCTs which were assessed as showing low risk of bias [23]. A further four reviews presented evidence from primary studies which could be considered of moderate quality overall [7, 21, 24, 25]. Three reviews were considered high-quality systematic reviews, but presented low quality evidence from primary studies [8, 9, 22]. In two of these reviews, most primary studies were cross-sectional in design with a high risk of bias [8, 9]. Five reviews presented evidence from primary studies where the quality was either not reported or not clear to the reader [2, 20, 26–28].

**Table 1:** Quality Assessment of Included Systematic Reviews

Author, year	Is there a well-defined Question?	Is there a defined search strategy?	Are inclusion/exclusion criteria stated?	Are the primary study designs and number of studies clearly stated?	Have the primary studies been quality assessed?	Have the studies been appropriately synthesized?	Has more than one author been involved at each stage of the review process?	Overall score (out of 7)
Cornish et al., 2014) [7]	Yes	Yes	Yes	Yes	Yes	Yes	Unclear	6
Prost et al., 2014) [23]	Yes	Yes	Yes	Yes	Yes	Yes	Yes	7
Skevington et al., 2013) [20]	No	Yes	Yes	Yes	Unclear	Yes	Unclear	4
Farnsworth et al., 2014) [2]	No	Yes	No	Yes	No	Yes	Unclear	3
Atkinson et al., 2011 [25]	No	Yes	No	Yes	Yes	Yes	Unclear	4
Kerrigan et al., 2013 [8]	Yes	Yes	Yes	Yes	Yes	Yes	Yes	7
Kerrigan et al., 2015 [9]	Yes	Yes	Yes	Yes	Yes	Yes	Unclear	6
Nachega et al., 2016 [21]	No	No	Yes	Yes	Yes	Yes	No	4
Musa et al., 2014 [27]	No	Yes	Yes	Unclear	Yes	Yes	Unclear	4
Gilmore and McAuliffe, 2013 [24]	No	Yes	Yes	Yes	Yes	Yes	No	5
Medley et al., 2009 [22]	Yes	Yes	Yes	Yes	Yes	Yes	Yes	7
Salimi et al., 2012 [28]	No	Yes	Yes	No	Yes	No	Yes	4
Okwundu et al., 2013 [26]	Yes	Yes	Yes	Yes	Yes	Yes	Unclear	6
Total	6/13	12/13	11/13	11/13	11/13	12/13	4/13	

### Key findings

**Fig. 2:**
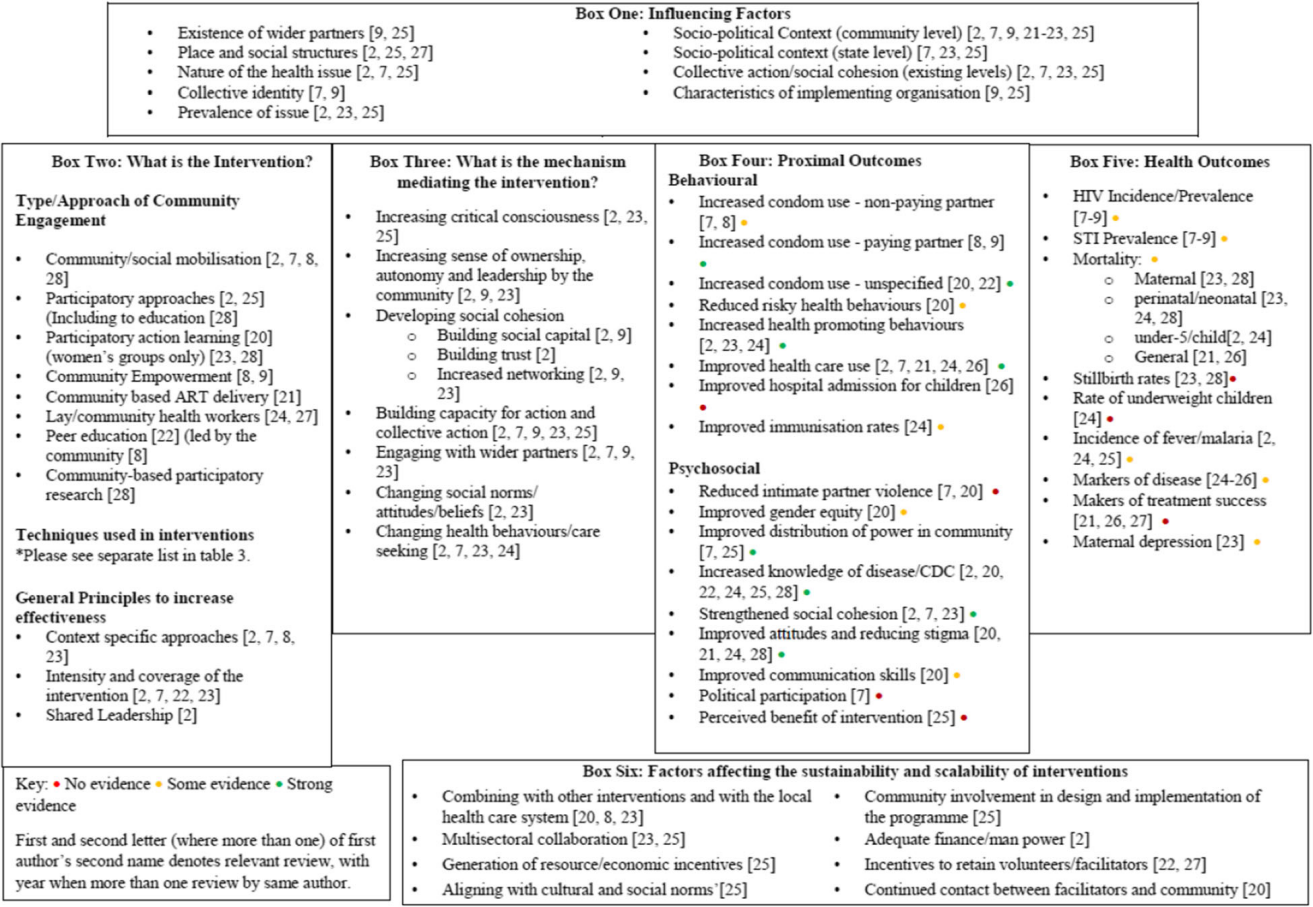
Synthesis of results showing intervention characteristics, mechanisms and outcomes

Figure 2 synthesises the results from the review of reviews in a format adapted from that of the MRC for complex interventions. The figure displays: i) influencing factors external to the intervention which impact on effectiveness (box one); ii) the types/approaches to CE, techniques used within the interventions and general principles identified that are integral to the design of the intervention (box two); iii) mechanisms mediating the intervention (box three); iv) the proximal (behavioural and psychosocial) outcomes (box four); v) final health outcomes (box five) and vi) factors affecting sustainability and scalability of interventions (box six).

#### Community engagement approaches and techniques

The reviews studied a range of CE approaches, often using broad terms such as community mobilisation, social mobilisation or community empowerment [2, 7–9, 25]. More specific approaches included the use of interventions delivered by community members or lay health workers (LHW) [21, 24, 26, 27] community based participatory research [28] and peer education [22].

Table 2 gives the main approach to CE taken by each review, the definition of the approach as provided by review authors and the health topic explored. As these descriptions suggest, communities were on the whole actively involved in the design, delivery or content of the interventions rather than being passive recipients of information only. Detail was lacking to evaluate the actual degree of ‘citizen control’ [29] across the interventions as reviews did not formally categorise their included interventions using any empowerment models.

**Table 2:** Community engagement approaches as reported in included systematic reviews

Health topic	Review	Community engagement approach	Definition (if provided by review, the actual text from the review is reported here)
HIV	Skevington et al., 2013, p1026 [20]	Participatory community intervention	Participatory learning approach to empower women and men to enhance control over their sexual and emotional relationships within the prevailing socio-cultural, economic and political context. Peer groups divided by gender and age-band (young/old) work separately, then together intensively over 3–4 months to build sexual health knowledge and reflect on behavioural motivation. The community analyses factors that mutually affect their lives and behaviour, and different generations of men and women engage with implementing positive change that could reduce HIV/AIDS vulnerability in their life and community
	Cornish et al., 2014, p2111 [7]	Community mobilisation	“For the purposes of this review, we take the term ‘community’ to refer to collective resources that exist among a community, rather than at the individual level. We take the term ‘mobilisation’ to mean capitalising on those community connections and strengths to generate new possibilities of action”. “Community mobilisation is considered as a component of externally-triggered HIV interventions, rather than including indigenous CM initiated by grassroots actors with broader interests than HIV”.
	Kerrigan et al., 2013 [8]	Community empowerment (FSW)	Empowerment, community mobilization intervention, empowerment of sex workers, Collectivization activities, Empowerment intervention activities
	Kerrigan et al., 2015 [9]	Community empowerment (FSW)	Empowerment, community mobilization intervention, empowerment of sex workers, Collectivization activities, Empowerment intervention activities
	Nachega et al., 2016, p4 [21]	Community based interventions	“Models could include the following: (1) home-based interventions (e.g., friends or family-centred approaches); (2) peer- or HIV patient-led interventions; community ART distribution points (with or without involving primary level formal or informal health facilities); (3) community-based ART adherence clubs (with or without involving primary level formal or informal health facilities); (4) community ART groups”
	Medley et al., 2009, p2 [22]	Peer education interventions	“the sharing of HIV/AIDS information in small groups or one-to-one by a peer matched, either demographically or through risk behaviour, to the target population. This definition distinguishes peer education from mass media programs that may be hosted by a peer, but where no interpersonal interaction occurs and information flows in only one direction”.
Malaria	Atkinson et al., 2011, p3 [25]	Community participation	A range of different interventions are included in this study. The authors advocate that communities are best placed to define what is meant both by ‘community’ and ‘participation’. However, two broad approaches have been previously described: vertical or ‘top down’ approaches, and horizontal or ‘bottom up’ approaches- pros and cons are identified with each.
	Okwundu et al., 2013, p6 [26]	Home or community-based programmes	“Any programme which trains mothers or caregivers, community-based volunteers, community-based health workers, or drug sellers to recognise and treat fevers with antimalarials presumptively or after a positive malaria RDT”.
TB	Musa et al., 2014, 104 [27]	Community based interventions	Use of lay community members to facilitate delivery of TB care. A lay health care worker is a member of the community, often without formal training in health care delivery, chosen by the community for the purpose of delivering some care needs. They are identified with other names such as community health care workers, community health care aides and village health care workers
Child and maternal health	Farnsworth et al., 2014, p69–70 & 79 [2]	Community engagement	Community participation and CE - specifically collaborative and shared leadership types of CE The authors use the term community mobilization to describe highly engaged, community-centred processes designed and implemented with the intent of improving a health outcome through a process of increased community capacity. “The Collaborate category applies to programs that form a partnership with the community on several aspects of the intervention including planning and management of the program. The highest step in the CE continuum is Shared Leadership, where final decision-making authority for the program is held by the community itself”. “A Shared Leadership categorization is determined by a strong bidirectional relationship between the program and the community and may include approaches initiated by the community itself. This relationship extends beyond communication to joint planning, implementation and ultimately approval on intervention elements. The Shared Leadership community intervention relationship includes the presence of strong partnership systems and structures between entities”
	Prost et al., 2014 [23]	Women’s participatory learning and action groups	The intervention mobilises communities (defined as individuals linked by shared concerns) concerned about maternal and child health (MCH) to take action by organising them into women’s groups and facilitating a four-stage participatory learning and action cycle.
	Gilmore and McAuliffe, 2013, p3 [24]	Community health workers (CHW)	Lay health care delivery - in this case by community health workers (CHW). Community health workers are defined here as ‘members of the communities where they work, should be selected by the communities, should be answerable to the communities for their activities, should be supported by the health system but not necessarily a part of its organization, and have shorter training than professional workers”
Birth related infection control practices STIs with a focus on HIV/AIDs	Salimi et al., 2012, p387 [28]	Community-based Participatory Research (CBPR)	Focus is on community-based participatory research (CBPR). “This kind of research aims to promote health or decrease inequality in health by attracting community participation...” “The emphasis of CBPR is on its participative process, which empowers main partners”.

A range of different techniques for community engagement were extracted from the reviews, however detail was generally sparse, with the exception of three reviews which provided greater information [2, 7, 25]. Frequently mentioned techniques included ‘sensitisation’ with the community, (e.g. raising awareness of a health intervention with the community before the intervention begins; allowing opportunity for engagement), as well as community members becoming directly involved in the delivery or organisation of health services.

Local knowledge and skills were utilised in other ways in some interventions- for example, involving the community to identify useful resources, individuals or issues, or engaging the community in the development of the intervention (e.g. through design of key materials or messages). Community members were also involved in the formation of groups, including participatory action cycles with women.

The community engagement techniques were often supported by external agents such as academic institutions or NGOs that provided training to volunteers or delivered equipment used in the interventions. Some interventions incorporated existing health structures, for example to employ supervision processes.

Table 3 outlines the main techniques identified across the reviews.

**Table 3:** Community engagement techniques and approaches found in the systematic reviews

Technique	Review
Sensitisation with the community, e.g. Raising awareness of a health intervention with the community before the intervention begins; offering opportunity for engagement.	[2, 7–9, 20, 22, 24–26]
Consultation with community leaders/members/stakeholders	[2, 7, 8, 25, 28]
Involvement of the community in identification /mapping of • ‘social actors’ e.g. local agents or organizations with resources • community members to deliver or promote interventions • positive behaviours/ good examples e.g. positive deviance methods • problems and priority setting	[2, 7, 23, 25]
Strengthening links to health systems or health service delivery e.g. lay person facilitation of health planning groups.	[2, 7, 8, 21, 23–27]
Community delivery of interventions, either in the household, via groups, via CM events, often using health education	[2, 7, 9, 21, 23–27]
Participatory learning and action cycle	[2, 7, 20, 23]
Formation of groups in the community	[2, 7, 20, 22, 23, 28]
Development of the community intervention, or aspects of it e.G. *key* messages/materials	[2, 25, 28]
Creating safe space for debate and conscientisation	[7, 9]

#### Outcomes and effectiveness

Within the reviews there was considerable heterogeneity in the types of primary studies included, and the outcomes measured. Table 4 summarises the main findings from each systematic review in relation to the impact of community engagement interventions on communicable disease outcomes. The results are collectively discussed in the following section. Outcomes have been categorised into impacts on mortality, disease incidence and prevalence, healthcare adherence and use, health literacy, health behaviour and psycho-social outcomes based on measures that were commonly reported across the reviews. A summary of the strength of evidence is featured in Box 4 and 5, within Fig. 2.

**Table 4:** Summary of quantitative outcomes from review papers

Author	Summary of key communicable disease control outcomes from community engagement interventions in low and lower middle income countries
	**HIV**
Cornish et al. [7]	*High risk populations (including FSW and MSM)* - Of three studies measuring HIV prevalence following community mobilisation interventions, one case control study showed that greater programme intensity was significantly associated with a lower HIV prevalence in three of six Indian states tested. In a cohort study of the same programme, CMI were associated with a significant reduction in the prevalence of HIV, whereas a further cohort study found no significant reduction in HIV. -Of three studies measuring the impact of CMI on other STIs, one showed a significant reduction in syphilis, and chlamydia and/or gonorrhoea in FSW, while another showed a significantly lower likelihood of HSV-2 and syphilis in FSW and MSM. A further study showed a significant increase in the prevalence of HSV-2, alongside significant decreases in syphilis, trichomonas, chlamydial infection and gonorrhoea. -Significant increase in condom use in four CMI studies with FSW, (although increases were non-significant under certain circumstances in two of these studies). Mixed evidence on condom use in MSM following CMI. -One study showed a significant increase in social support but not political participation following a community mobilisation intervention. -Significant association between being a member of a help group and experiencing higher perceived collective efficacy and support in one study. *Youth population* -Four studies showed non-significant effect of CE intervention on HIV incidence or prevalence. - Effects on the incidence/prevalence of other STIs were mainly non-significant; one study showed a significant reduction in gonorrhoea and syphilis incidence following the CE intervention. Another study showed a significant decrease in HSV-2 incidence. -One study showed a significant increase in the rate of condom use with casual partners, however four studies showed no significant changes in condom use with regular partners. -HIV testing was significantly increased in one intervention using community based voluntary testing compared to standard care.
Skevington et al. [20]	-Condom use significantly increased in two of five studies following the ‘Stepping Stones’ (SS) CE intervention. No significant changes were seen in the other studies. -Of the two studies that reported the effect on multiple sexual partners, one showed a significant reduction following the intervention. -Two of five studies showed a significant decrease in alcohol use before sex following the CE intervention. One study showed that communities participating in SS used significantly less alcohol than non SS villages. -One of five studies reported a significant increase in individual knowledge following the SS intervention, another study showed a significant increase in knowledge at a community level compared to non-participating villages. -Of two studies measuring changes in gender equity, one study showed significant improvements in some attitudes following the intervention. -Two of five studies reported improvements in attitudes towards those living with HIV and AIDs following the SS intervention, one of which reported statistical significance.
Kerrigan et al., 2013 [8]	-Two of three studies measuring HIV infection showed an odds ratio that was significantly protective in favour of the community empowerment intervention at a follow up of 2.5 years. - In meta-analysis of three studies, community empowerment was associated with decreased odds of gonorrhoea but not chlamydia. - Condom use was measured in six studies. Five studies showed that community empowerment was associated with significantly higher odds of condom use with clients, however there was statistical heterogeneity in this result. -Three studies measured consistent condom use with regular non-paying partners and no significant associations with CE were found.
Kerrigan et al., 2015 [9]	All relevant results are from community empowerment studies conducted in India; -Results from nine intervention sites were combined in meta-analysis and showed a significantly reduced prevalence of HIV in sex workers following the community empowerment intervention (heterogeneity was high). -Meta-analysis of results from four intervention sites showed a significant reduction in the odds of syphilis. -Of ten intervention sites measuring the impact of community empowerment interventions on gonorrhoea prevalence, five showed significantly reduced odds of gonorrhoea. -Similarly, of ten intervention sites measuring the impact on chlamydia risk, four showed significant reductions in the odds of chlamydia, (five showed non-significant reductions and one showed a significant increase in the odds of chlamydia). -Condom use was measured in one RCT and showed a significant improvement over time in intervention participants compared to controls. -Meta analysis of results from cross sectional studies over six intervention sites showed significantly increased condom use with regular clients (heterogeneity was high). -A further seven and five intervention sites reported significant increases in condom use with all clients and condom use with new clients respectively.
Nachega et al. [21]	-Of seven RCTS and two cohort studies measuring the impact of community-based delivery of antiretroviral therapy (ART), one RCT showed a significant decrease in all-cause mortality in the intervention group compared to control group. The remaining studies showed no significant differences between groups. - Virologic suppression at 12 and/or 24 months after ART initiation was measured in six RCTS and two cohort studies- no significant differences between the intervention and control groups were found. -Two of five RCTS that measured optimal ART adherence levels showed a significant increase following the community-based initiative, while three showed a non-significant reduction in adherence levels. -Six RCTs and two cohort studies measured retention in care, and no statistically significant differences were found between those receiving the community-based initiative and those in the control group.
Medley et al. [22]	-Of five studies measuring the impact of peer education on STI infection, one study showed a significant decrease in STI infection, one showed a significant increase in STI infection and three studies showed non-significant reductions in STI risk. - Of ten studies reporting the impact of peer education on condom use, five showed significant increases in the likelihood of use, four showed non-significant increases in the and one showed a non-significant decrease in the likelihood of condom use. -Of ten studies, seven showed a significantly positive impact on HIV knowledge associated with peer education interventions.
	**MALARIA**
Atkinson et al. [25].	*Eight studies provided quantitative results;* *Biological outcomes* -One community engagement intervention showed a statistically significant reduction in prevalence of STD symptoms in the intervention compared to control groups. - A community engagement intervention for the treatment of malaria showed a significant reduction in mean incidence of malaria per 10,000 person weeks over 2 years compared to control. *Behavioural* -A community delivered intervention showed significantly increased coverage for vitamin A supplementation, bed nets and anti-malaria treatment compared to control districts, however no significant difference was found in directly observed therapy (DOT) between the intervention and control areas. - In a study of lymphatic filariasis, no significant difference was found in drug distribution and consumption when this was devolved entirely to communities or delivered routinely by medics. - A study of environmental modification plus community participation showed significantly higher perceived benefits of drain cleaning in the intervention communities compared to the control group (61% vs 30%). *Psychosocial* - A study of health and feedback committees in communities in Cambodia found engagement of existing community-based structures more effective for community participation than externally introduced structures. - A community directed intervention (CDI) approach using traditional kinship systems for the treatment of onchocerciasis showed significantly better disease knowledge, significantly lower control by leaders and increased treatment coverage compared to a standard CDI approach.
Okwundu et al. [26].	*Ten studies were included in this review;* -One trial showed a significantly reduced risk of mortality in the home or community-based programme compared to facility-based care. - Two trials to measure parasitaemia showed mixed results- one showing a significantly reduced risk in the intervention group, the other not. - Evidence from one trial showed no significant impact on hospitalisation for children, when mothers had been trained to treat fevers. - Pooled results from two trials showed a significant increase in prompt treatment with anti-malarials in the intervention group, compared to control. -The pooled results of two trials showed that the use of rapid diagnostic testing compared to clinical diagnosis in community-based programmes reduced prescribing of antimalarials however there were no differences in hospitalisation or all cause morbidity.
	**CHILD AND MATERNAL HEALTH**
Prost et al. [23].	-Meta-analysis of seven RCTS showed exposure to women’s groups was associated with a 23% non-significant reduction in maternal mortality, a 20% significant reduction in neonatal mortality and a 7% non-significant reduction in stillbirth, with significant heterogeneity for maternal and neonatal results (NB these results represent all-cause mortality). -Five of seven studies measured ‘increased handwashing by attendants before home deliveries’: Of these five studies, there was a significant difference between intervention and control groups in three studies. -Four of seven studies reported increased use of clean delivery kits for home births. Of these four studies, three found significant differences between intervention and control groups.
Farnsworth et al. [2].	*Communicable disease specific outcomes were measured in five studies:* *Biological outcomes* - Significant decrease in child deaths due to malaria in one study using an eight stage CE intervention. - Reduced prevalence of fever in relation to community-based control of malaria, in one study. *Behavioural outcomes* -Improved hygiene in birth delivery practices in one study following a range of collaborative approaches and CE techniques. - Two studies showed significant increases in net use for malaria prevention following a community engagement intervention; Another study showed significant increases in water disinfectant use with a study utilising volunteer health promoters to deliver motivational interviewing. -Increased care seeking for malaria was found in one study that followed a health promotion approach with participation, empowerment and contextualisation. *Psychosocial outcomes* - Two studies showed improvements in knowledge following community engagement interventions, in the areas of malaria knowledge and water disinfectant use. - Social cohesion was increased in two studies, alongside increases in social capital and trust following CE interventions. Collective self-efficacy (community empowerment) increased in three studies.
Gilmore et al. [24]	*Biological outcomes* -Of five studies measuring the impact of community health worker programme on rates of diarrhoea, four showed significantly reduced rates of diarrhoea in infants or children, two using educational approaches, one through breastfeeding promotion and one through the promotion of Kangaroo care. Another breastfeeding intervention showed no significant difference in the prevalence of infant diarrhoea in the intervention and control group, despite demonstrating significantly higher breastfeeding rates. -One study of CHWs reported a reduction in under 5 year mortality rates of 53%, at 18 months following the intervention (no tests of significance provided). The same study reported that malaria and or fever prevalence was significantly reduced by 5.8% in the intervention group. - A trial using CHWs to promote DPT-3/Hep B vaccination demonstrated that full immunization rates were 32% higher in the intervention group at 4 months. *Behavioural and psychosocial outcomes* -A further study of CHWs in antimalarial treatment and bed net distribution reported significantly higher rates of bed net use in pregnancy and rates of antimalarial treatment in the intervention group compared to the control. -In a study of CHWs in an urban slum, poor sanitation and hygiene practices were significantly reduced in the intervention group compared to the control. In addition, there was significant improvement in mother’s knowledge, attitude and practice regarding diarrhoea etiology and sanitation and hygiene.
Salimi et al. [28]	Three relevant studies were included in this review of community based participatory research; -One cluster RCT, using a participatory learning and action cycle with women’s groups in Nepal showed a significant reduction in neonatal mortality and in maternal mortality rates in the intervention group compared to the control over 2 years. There were no significant differences in stillbirth rates. -A longitudinal, experimental study using participatory action research (PAR) with high risk heterosexual males in the Philippines showed significant increases in condom use and attitude towards condom use at post-test and 6 months compared to baseline. The reported STI incidence also decreased significantly at post- test and 6 months’ time points. -A further cluster RCT using participatory approaches with community leaders to promote a healthy living environment showed a significant increase in scores relating to ‘healthy living environment competencies’ following the intervention. These competencies were in areas such as sanitation, hygiene and prevention of diseases. No significant changes in these competencies were seen in the control group.
	**TB**
Musa et al. [27].	- Pooled outcome from five studies shows no significant difference in TB treatment success when TB care was delivered by lay health workers compared to facility-based care. However, stratified analysis of a small number of studies showed that LHW interventions in rural settings significantly increased TB treatment success compared to standard facility-based care with no significant difference in urban studies.

#### Mortality

Overall, some measure of mortality was reported in six of the reviews. In a review of women’s groups practicing a participatory learning and action approach focused on child and maternal health, Prost found a trend towards a reduction in maternal mortality and still births on study data combined with meta-analysis, although neither result reached significance. The same review did however find a significant 20% reduction in neonatal mortality on meta-analysis [23]. Despite this result representing ‘all cause neonatal mortality’ rather than communicable disease specific mortality, the authors theorise that the reductions in neonatal mortality may have been due to improvements in hygiene [23].

In other reviews looking at child and maternal health, a significant decrease in child deaths due to malaria was found in a single study using an eight stage CE intervention [2] and a single community health worker intervention reported a 53% reduction in under 5 year mortality, although statistical significance was not stated in the review [24]. Similarly, a single study within Salimi [28] using a participatory learning and action cycle with women’s groups showed a significant reduction in maternal mortality and also in perinatal mortality. In a review of home or community-based programmes for treating malaria in rural Ethiopia, one of ten primary studies from LLMICs demonstrated a significantly reduced risk of childhood all-cause mortality in comparison to facility-based care [26].

Measures of risk reduction in mortality were less commonly reported in interventions targeted at adults; One review measured this outcome for patients living with HIV that had received an intervention of community-based ART delivery [21]. Of six RCTs and two cohort studies only one RCT demonstrated a significant reduction in all-cause mortality [21].

Overall, the evidence for effectiveness of CE interventions to reduce mortality is mixed. There does seem to be better effectiveness for child or maternal mortality compared to general mortality.

#### Disease incidence and prevalence

Significant reductions in HIV prevalence were demonstrated in meta-analyses of two review papers of empowerment interventions with sex workers, although study quality was often low and heterogeneity high [8, 9]. There was considerable overlap in the primary studies within these reviews. A further review found more mixed inconclusive evidence to support an impact on HIV prevalence in sex worker populations and mostly non-significant effects on HIV prevalence in youth and general populations [7].

Community empowerment approaches for female sex workers were associated with significantly decreased odds of gonorrhea, chlamydia and syphilis [9]. An earlier review by the same authors noted a trend without always reaching statistical significance in these fields [8]. Cornish et al. [7] found some evidence of a positive impact of community engagement interventions on STD prevention in sex workers but found little evidence for this effect within the general or youth population. Peer education approaches found mixed effects on STI prevalence (including one study that saw a significant increase in STI infection [22]. Salimi [28] reported a significant decrease in STI rates within a community based participatory research paper, however this was a finding from a single study within the review. Overall, the evidence for reduction of STI prevalence through CE interventions is mixed. It is strongest within reviews focused on sex workers [8, 9] and in high risk populations [7], although the risk of bias in the primary studies within some of these reviews was high [8, 9].

Three reviews showed a significant reduction in fever or malaria incidence or prevalence following community engagement methods; two used a generalized CE approach [2, 25] the third used community health workers [24]. However, this finding only came from one primary study within each review.

A range of health outcomes which, broadly speaking are markers for disease outcome were also reported in some reviews. A positive difference was shown (STD symptoms, prevalence of infant diarrhea, microbial load) with a combined approach of general community engagement and use of community health workers approaches [24, 25]. In a review of community health worker interventions, four out of five studies reported a significant reduction in infant or child diarrhoea; studies were rated as moderate quality [24]. In a different review, a home/community-based approach showed no significant difference in rates of anemia (meta-analysis) and parasitemia (individual studies) [26].

#### Markers of healthcare adherence, success and use

Delivery of treatment for communicable diseases using community approaches was reported in three reviews; a review of community-based delivery of ART for HIV found no significant impact on virological suppression and mixed results regarding the impact of the community intervention on optimal ART adherence levels, in comparison with facility-based care [21]. Mortality was also generally not reduced following this intervention (as reported above) [21]. This review included results from seven RCTs with a generally low risk of bias.

Pooled results from five studies of TB treatment success showed no significance difference in outcome when TB care was delivered by lay health care workers compared to facility-based care overall, but when stratified for urban vs rural environment a significant improvement was found in the rural setting; within the urban environment the difference remained non-significant [27]. A single study within Atkinson et al. that compared community-based treatment of lymphatic filariasis to standard medical care showed no significant difference in drug distribution and consumption when this was devolved entirely to communities, rather than delivered by medics [25].

Overall, on the basis of the reviews that were captured within this systematic review, the evidence does not generally appear to support an impact of CE interventions to improve medical treatment delivery for communicable disease control and management in comparison with facility-based care, although there is great variation in the types of approaches and studies reviewed.

Where community engagement methods were used to encourage ‘health care use’ positive outcomes were reported across several reviews [2, 21, 23–26]. Significant differences were reported in attending for first HIV test, treatment engagement, prompt treatment of fever and reduced prescribing of anti-malarials. No significant difference was found in retention of care for those with HIV treated with a community -based initiative compared to standard care [21]. An increase in care seeking behaviour was observed in two reviews [2, 23] but no effect was found in a third [7]. A single trial of community health workers to promote DPT-3/Hep B vaccination demonstrated that full immunization rates were 32% higher in the intervention group at 4 months [24]. Prost [23] reported on service accessibility and quality in a review on women’s group led participatory action cycles and found that the interventions helped groups to take action to improve these factors. Overall, there is convergent evidence that CE interventions can impact on health care use.

#### Behavioural and health literacy outcomes

Consistent evidence for significantly increased condom use following participation in community engagement interventions comes from four studies [7–9, 20]. A stronger effect was noted amongst individuals with partners that paid for sex than individuals with non-paying partners.

Health risk behaviours were assessed in one review taking a participatory learning approach [20]. Mixed effects were found in reduction of multiple sexual partners in two studies, however positive effects were found in reducing alcohol consumption in intervention communities [20]. HIV testing was measured in one study within Cornish et al. significant improvements were found [7].

Significant improvements in preventative behaviours such as bed net use, water disinfectant use and clean delivery practices, sanitation and hygiene practices and breast feeding were found in several reviews [2, 23, 24] taking general CE, community health workers and participatory learning and action approaches. Whilst most of the evidence for these outcomes comes from single studies, overall it provides convergent evidence that CE interventions may be effective in promoting communicable disease preventative behaviours.

Knowledge of disease or communicable disease control was reported within six reviews [2, 20, 22, 24, 25, 28] and all reported some increases in knowledge due to the intervention across all types of CE approach. This included one meta-analysis [22].

#### Psycho-social outcomes

No significant difference was found in the reduction of intimate partner violence [7, 20] and the evidence for a difference in gender equity was mixed in the one review which reported it [20]. Significant differences were found for improved distribution of power in the community [7, 25]. For example, a community delivered initiative that used a traditional kinship approach compared to a standard CDI for the treatment of onchocerciasis found significantly lower levels of control over decision-making by leaders [25].

Significant improvements were found in perceived stigma [20, 21] and attitudes towards those with communicable diseases [20, 28] although these were only reported in a small number of individual studies with no pooling of data within the reviews. Social cohesion was reported within two reviews, in both it was increased though details were limited [2, 23]. Social capital was also found to be increased in two studies within Farnsworth [2]. Collective efficacy (community empowerment) was shown to have increased in four studies taken from two reviews [2, 7]. Trust was measured in two studies within Farnsworth [2] and found to have increased following the community engagement intervention. Overall, there is some evidence that CE interventions can positively influence these key concepts within CE.

### Mechanisms through which CE influences communicable disease outcomes

The proposed mechanisms through which CE interventions were found to lead to improvements in communicable disease control and management are shown in box three within Fig. 2. These were not explored equally within the reviews and so the following mechanisms are based on only those reviews which did discuss them [2, 7, 9, 22–25].

Community engagement mechanisms were shown to act at various levels- for example at an individual level through encouraging health behaviour change, at a family level through actions such as increased child vaccination, at a societal level, or with external agencies. Mechanisms acting at a community or societal level were mentioned frequently; aspects of this included developing social cohesion (for example through increased networking and building trust), as well as generating increased capacity for action [2, 7, 9, 23, 25]. Prost [23] theorised that women’s learning and action groups acted as the catalyst to enable communities to better organise themselves, and from this take action on multiple aspects of health. In a review of CE interventions for child health and development, Farnsworth et al. [2] described that following knowledge acquisition, it is the new norms, levels of cohesion and self-efficacy that helps communities to achieve behaviour change. Developing a sense of ownership, increased autonomy and encouraging leadership within the community were also identified as key elements of CE interventions [2, 9, 23, 25].

The vast majority of identified mechanisms came from the same few reviews [2, 7, 9, 23, 25]. These reviews had either a general or a participatory learning approach to CE interventions, meaning the findings reported in this section may not be applicable to other approaches such as lay health care workers or peer education.

### Influencing factors - contextual and population level

The importance of the socio-political context in supporting or hindering community engagement was recognised, both at a community and state level. At a community level, characteristics such as stigma, the marginalisation of some groups and uneven power structures, particularly with regard to gender equality were identified as impacting the effectiveness of interventions, generally acting as barriers to success [8, 9, 22, 25]. At a state level, policies and laws can have a strong impact on participation in CE interventions [9, 25]. The political environment within a country can influence the degree of collectivism within society and associated concepts such as community spirit and trust, which may would normally be supportive of increased community participation in initiatives for health [25]. Policy decisions both nationally and internationally can influence allocation of funds, and the importance of community engagement practices within a health system [9, 25]. On a more local level, laws against certain practices (such as sex work) can deter individuals from being able to openly network and organise collective action [9]. As such, taking account of the socio-political context was highlighted as an important component of CE interventions [7, 9, 20]. Wider partners can exert a positive influence, for example if non-governmental organisations are in a position to lobby for improvements in the community [25]. However, there is the potential for them to inhibit effectiveness if they are not supportive of CE interventions [8, 9, 25]. The characteristics of the implementing organisation were also found to influence engagement and participation [25], with greater engagement seen when an implementing organisation supports rather than directs [9]. Using pre-existing services or organisations already situated within the community may have more legitimacy than a new organisation created for the purpose of CDC [25].

Place and pre-existing social structures also impacted on effectiveness; multiple reviews found CE interventions were more effective in rural than urban locations due to pre-existing social networks in rural locations and poorer initial population health for the diseases addressed (and hence greater scope for improvement) [2, 25, 27]. The local infrastructure and geographical accessibility were also important influences on participation [25] . The nature of the health issue (for example, pre-existing beliefs, misinformation) impacted on effectiveness, as did the extent or prevalence of the health issue, with prevalent diseases more likely to trigger participation in engagement activities [25].

The pre-existing level of collective identity, action and social cohesion also impacted on the effectiveness of the intervention; with high pre-existing levels being associated with successful CE interventions [2, 7–9, 23, 25]. Atkinson et al. (2011) provided a detailed overview of factors influencing participation in communicable disease interventions, the full scope of which cannot be captured in this review [25].

A general trend was found across several reviews for a greater impact of CE interventions on certain population groups, including sex workers, MSM and women’s groups, and some reviews theorised this may be linked to a stronger collective identity in these groups [8, 9].

A key principle shared by most of the interventions within the Kerrigan reviews was the stimulation of sex worker’s individual and collective identity to address inequitable social structures [9]. Cornish [7] states that one of the main characteristics of interventions aimed at sex workers and at- risk groups is that they capitalise on the collective identity of the group. They further suggest that interventions are more effective in groups with a meaningful collective identity; this may occur in different ways, for example through a stronger sense of cohesion as a group, and if there are specific structural barriers that the group may attempt to overcome (eg policies and laws that deter sex work) [7]. Members of the general population may not display the same marked disadvantages as marginalised groups and so the need is not always recognised to tackle the social determinants of their situation [7]. It was also recognised it was plausible that mobilising a sub-set of a population is easier than mobilising an entire community [7].

With regard to marginalised groups, Atkinson [25] explored the effect of vulnerability in depth (almost half the papers included in that review referenced the effect of vulnerability on participation). Whilst vulnerability can be a barrier to participation in health interventions, when empowered, action can be taken to reduce vulnerability. Atkinson suggested the most effective way to do this is to facilitate the self-identification of community problems, provide support to develop their own solutions, releasing latent capacity and improving community resilience.

### General principles identified within the CE approaches to increase effectiveness

#### Being aware of, and responding to context

The importance of tailoring the approach to the specific context was highlighted across many of the reviews [2, 7–9, 20, 23, 25]. The contextual and evolving nature of CE interventions was described in Cornish: ‘CM is, by its very nature, contextual and evolving. CM mobilises contextually-specific local networks, in locally-appropriate ways, and allows communities power to create and alter objectives. Thus, CM is not simply an intervention that is equivalent across sites, but takes different forms in different sites.’ [7], (Pg 2131).

Making the intervention specific to the particular context was an important influencing factor in success – including ensuring the intervention is acceptable; and recognising the local social and political context and existing beliefs and knowledge about the health topic and using these to reduce barriers to participation [25].

#### Intensity and coverage of target population

Intensity and coverage of the intervention were found to be important to the overall effectiveness in several reviews [2, 7, 22, 23]. A greater effect was found with greater intensity of the intervention, when assessed by some outcome measures [2, 7]. For example, a review on generalised community mobilisation approaches to HIV prevention found that the strength of the intervention had a significant effect on HIV prevalence, collective identity, collective efficacy and collective agency, and odds of violence or abuse [7].

#### Shared leadership/decentralisation/ ability to control

Shared leadership or a sense of ownership is seen to be of importance to the effectiveness of CE interventions [2, 9, 23, 25]. Kerrigan [9] concludes ‘the community empowerment process should be envisioned, shaped, and led by sex workers themselves if it is to be effective and sustainable in reducing sex workers’ risk for HIV and promoting and protecting their health and human rights’ (pg 179).

Decentralisation of the decision-making process to the local level was found to reduce resistance and improve participation in CE intervention [9, 25]. This may be a direct effect, or may act through ensuring that the community has an influence over the intended target of the intervention [2]. One review found that implementation of the intervention must be within the capacity of the community, if the community is to be motivated to engage [25] and another found that community engagement interventions tended to be more effective on behaviours over which the community has direct control, such as home hygiene, household nutrition or visits to antenatal care [2].

## Discussion

This review aimed to provide an overview of the evidence of effectiveness for community engagement interventions for communicable disease control in low and lower-middle-income countries. Across the included reviews we found that CE can significantly reduce neonatal mortality, HIV and other STIs, malaria incidence, and diarrhoea. Some other studies which, due to the methodology employed, were not included in this review confirm this finding. For example, a study in Ethiopia showed that peer-to-peer training of mothers significantly reduced child mortality in a holoendemic malaria area [30]. However, other studies suggest that CE approaches can have mixed impacts on health outcomes. A study from rural Guinea Bissau, which aimed to assess whether an intervention package that provided outreach services, trained community health workers, and delivered a community mobilisation strategy could reduce under-5 mortality, in an area where the health service infrastructure was very weak, found that the intervention package did not impact on child mortality, but did have an effect on maternal mortality [31].

In the included reviews, there was limited evidence that CE improvesmedical treatment uptake and adherence for communicable disease control and management. However, it is important to note that one of the included reviews is very clear that community-based approaches are “equal and certainly not inferior compared to facility-based ones and may in fact be superior when it comes to selected outcomes such as retention in HIV care”, [21, p7]. This finding is corroborated by other studies, which were not included in the reviews included in this review. For example, family-member direct observation of treatment (DOTS) for tuberculosis has been shown to be as effective as health worker DOTS [32], and globally, there is a notable emphasis on CE approaches in relation to tuberculosis treatment [33]. We suggest, therefore, that there are indications that CE approaches can be at least as good as other approaches in relation to improving treatment update and adherence.

While evidence for the impact on health outcomes was at times inconsistent, the effects of CE on proximal outcomes such as preventive health behaviours was evident across the included reviews. This finding is consistent with evidence from high-income contexts [10] which found positive impacts of community engagement on health behaviours, health consequences, self-efficacy and perceived social support outcomes, across both communicable and non-communicable conditions, but noted the insufficiency of the evidence base in determining impacts on longer-term health outcomes or on addressing inequities within communities.

Most striking was the impact of CE approaches on social outcomes such as strengthening bonds between individuals within communities, levels of trust and social cohesion [34]. This is consistent with evidence from high income contexts which has found stronger evidence for the effects of CE on the social determinants of health such as housing, crime, social capital and community empowerment than on health outcomes [10]. Given the strong evidence of the influence of social capital on health and [35] particularly the health of the poorest [36], it may be that more conclusive evidence of impacts of CE on health outcomes will be more apparent once social outcomes are well established. However, recent work to synthesise studies of the impacts of social capital highlights [37]] the importance of understanding context and improving the way context factors are recorded and reported within evaluations of complex public health interventions. These reflections are pertinent to this review where contextual factors, particularly the socio-political contexts, characteristics of implementing organisations and their partners, as well as the prevalence, nature and social norms around the health issue being studies, were identified as one of the principles underlying effective CE approaches.

Additional principles identified in this review were establishing shared leadership, decentralisation of decision making and an ability for community members to control the intervention. A realist synthesis of CE approaches in high-income contexts specifies a set of eight principles which focus on how to operationalise principles for CE by for example providing transparent leadership, trust, early engagement, shared decision-making and recognising power imbalance [38]. While these provide a helpful steer for those designing CE programmes, a more comprehensive assessment of the barriers and facilitators to CE comes from a review of UK-based CE studies which identifies three key areas which affect CE: context, infrastructure and processes [39]. The conscious translation of these broader principles into CE programmes in LLMICs has received further impetus from WHO with the development of a framework for community engagement. This focus was triggered by the Ebola outbreak of 2014 where transmission only began to slow once engagement and trust with communities had been established [40].

Our review emphasises that CE can impact on CD health outcomes, but this is often dependent on contextual issues related to the CD itself, shared identities, social capital and the institutional and socio-political context.

### Strengths and limitations

Umbrella reviews are naturally limited to the evidence that currently exists within systematic reviews in that topic. They may therefore, exclude important findings from individual studies which have not been synthesised in this way. This appears to be the case in terms of key areas such as TB, which had limited quality representation in systematic reviews of the community engagement evidence. Furthermore, in covering such a broad topic (in this case community engagement for communicable disease control), the reviews are likely to be heterogeneous. We found this to be the case in our research, with reviews varying in terms of the approach and definition of the intervention, the health topic targeted, the kinds of primary study included and the kind of analysis that has been undertaken. As a result, it was difficult to produce quantitative summaries. However, a narrative approach to data synthesis was found to be more viable.

Application of inclusion and extraction criteria was particularly difficult. For the majority of reviews, only a proportion of the primary studies within the review were relevant and so information relevant only to those primary studies could be extracted. This created difficulties when extracting narrative synthesis and conclusions from each review paper, as these were usually based on the entirely of primary studies. The results of meta-analysis also had to be interpreted carefully if not all the underlying studies were relevant.

One of the main limitations to umbrella reviews is the lack of detail in the underlying reviews. It is beyond the scope of most reviews of reviews to revisit the primary studies referenced, and so individual studies are only reported as in the original review. At each stage of review, detail about the original intervention is lost.

A specific limitation of our review is that, due to resource limitations within our study, we were unable to include non-English language reviews within our search. It should be noted that of the included reviews, seven had no language restrictions in their inclusion criteria, one only included English, Spanish and Portuguese papers and one only included English and French studies. In light of this, our umbrella review does include evidence from studies published in languages other than English, however we acknowledge that these studies, and the regions they focus on such as south and central America, China and Franco-phone and Luso-phone Africa may be underrepresented in our findings.

More broadly, there are methodological limitations that are associated with a review of reviews which limit their validity. In addition, many of the conclusions drawn (including many of those based on meta-analysis) are not on the basis of the results of RCTs. This limits the validity of such reviews: there is a need for further high quality primary research of interventions for a range of health problems, and subsequent systematic reviews. We also suggest all primary assessments of interventions are accompanied by a process evaluation that includes assessment of fidelity of implementation of the intervention, impact of context, scalability and sustainability.

### Implications for further research

When embarking on the umbrella review, we considered current ambitions to achieve the sustainable development goals for infectious disease control. By scoping such an extensive research area, we were able to identify gaps in the current research base which may be required to aid progress in this area. We found that the majority of systematic review literature on community engagement interventions for communicable disease control currently relates to HIV and/or other STI prevention and treatment (six reviews), with a further four reviews in the area of child and maternal health, two in malaria and only one in the area of TB treatment. We found no systematic reviews to describe the impact of community engagement interventions in new and emerging infections, or in the context of outbreak management. Our research did however highlight promising results in the use of community engagement interventions in marginalised groups, suggesting that for HIV/STI prevention, CE may be effective in engaging with these populations. Further research is required to investigate whether community engagement initiatives may be successful in the prevention and management of other types of infectious disease with marginalised populations. An area that was rarely explored in the reviews or the primary studies they included was the factors that affected the sustainability of CE programmes. Given the short-term nature of many programmes and research studies, this is to be expected. However, further emphasis on factors leading to sustainability would provide valuable evidence to inform the design of future CE programmes.

## Conclusion

Our review of CE demonstrates that CE interventions can be effective in contributing to CDC in low and lower-middle-income settings. Measuring impact on health outcomes is challenging within the resources available for research in low-income contexts; interventions seem to be more effective in improving behavioural and psychosocial factors. The use of a conceptual model showing the influence of context, and identifying intervention components, sustainability factors and mechanisms for change is helpful in identifying potential impact on health outcomes. The influencing factors, mechanisms, general guiding principles and factors for sustainability are all inter-related and support each other conceptually. These provide a good framework of factors to consider for those developing CE interventions, particularly within CDC.

## Supplementary information

**Additional file 1.** Search Strategies.

**Additional file 2: Table S1.** Characteristics of Included Systematic Reviews.

## Data Availability

None.

## Abbreviations

ART: Antiretroviral therapy
CD: Communicable Diseases
CDC: Communicable Disease Control
CE: Community Engagement
CHW: Community health worker
DARE: Database of Abstracts of Reviews of Effects
DOTS: Direct Observation of Treatment
FSW: Female Sex worker
HIV: Human immunodeficiency virus
LHW: Lay Health Worker
LLMIC: Low and Lower middle income countries
MRC: Medical Research council
MSM: Men having sex with men
OECD: Organisation for Economic Co-operation and Development
PRISMA: Preferred Reporting Items for Systematic Reviews and Meta-Analyses
RCT: Randomized controlled trial
SDG: Sustainable Development goals
SS: Stepping Stones
STI: Sexually transmitted infections
TB: Tuberculosis
UNAIDS: United Nations Programme on HIV and AIDS
UNDP: The United Nations Development Programme
WHO: World Health Organization
