# Supplementary material for: Community engagement interventions for communicable disease control in low- and lower- middle-income countries: evidence from a review of systematic reviews
Source: Int J Equity Health. 2020 Apr 6;19:51. doi: 10.1186/s12939-020-01169-5 (PMC7137248; doi:10.1186/s12939-020-01169-5)
Supplement: Supplementary file 1 — Additional file 1. Search Strategies. [file 12939_2020_1169_MOESM1_ESM.docx]

Search Strategies

# Project Name: Community Interventions

## Search 3b. Community engagement for diseases in LMICs – Systematic reviews

# Date: 9 June 2017

## Database: CAB Abstracts <1910 to 2017 Week 22>

**Search Strategy: CAB Community Engagement Disease LMIC Q3**

--------------------------------------------------------------------------------

1 developing countries/ (2031668)

2 (Low* income* adj3 (countr* or nation* or economy or economies)).tw. (2976)

3 (LIC* adj3 (countr* or nation* or economy or economies)).tw. (185)

4 ((Developing or underdeveloped or less-developed or "less* developed" or "third world") adj3 (countr* or nation* or economy or economies)).tw. (2047376)

5 ((Underserved or "under served" or deprived or poor*) adj3 (countr* or nation* or economy or economies)).tw. (4168)

6 ((Developing or "less* developed" or less-developed or "under developed" or underdeveloped) adj2 world).tw. (5408)

7 ("Transitional country" or "transitional countries").tw. (89)

8 Afghanistan/ (3211)

9 central africa/ (29592)

10 east africa/ (123731)

11 Angola/ (2601)

12 "Sao Tome and Principe"/ (456)

13 Bangladesh/ (24807)

14 Benin/ (4850)

15 Burkina Faso/ (7009)

16 Burundi/ (1414)

17 Cambodia/ (4356)

18 Cameroon/ (12130)

19 central african republic/ (1558)

20 Chad/ (2137)

21 Comoros/ (554)

22 Congo/ (3523)

23 Congo Democratic Republic/ (7465)

24 Djibouti/ (327)

25 Equatorial Guinea/ (557)

26 Eritrea/ (1035)

27 Ethiopia.gl. (21112)

28 Gambia/ (2870)

29 Guinea/ (1717)

30 Guinea-Bissau.gl. (873)

31 Haiti/ (2236)

32 Kenya/ (34382)

33 Korea Democratic People's Republic/ (942)

34 Laos/ (2846)

35 Lesotho/ (1430)

36 Liberia/ (1561)

37 Madagascar.gl. (8943)

38 Malawi/ (9777)

39 Mali/ (5407)

40 Mauritania/ (1162)

41 Melanesia/ (17493)

42 exp micronesia/ (2285)

43 exp "Federated States of Micronesia"/ (286)

44 Kiribati/ (297)

45 Solomon Islands/ (2087)

46 Tuvalu/ (199)

47 Mozambique/ (5361)

48 Myanmar/ (5933)

49 Nepal/ (13114)

50 Niger/ (3580)

51 Rwanda/ (3216)

52 Senegal/ (9406)

53 Sierra Leone/ (3170)

54 Somalia/ (2983)

55 South Sudan/ (205)

56 Sudan/ (13992)

57 Tajikistan.gl. (2780)

58 Tanzania/ (22801)

59 Togo/ (2638)

60 Uganda/ (16645)

61 Vanuatu/ (1304)

62 Yemen/ (2786)

63 Zambia/ (8413)

64 Zimbabwe/ (15886)

65 (Afghanistan* or Angola* or "Atlantic Islands" or Bangladesh* or Benin* or Bhutan* or "Burkina Faso" or Burma or Burmese or Burundi* or Cambodia* or "Central African Republic" or Chad or Comoros or Congo or Djibouti*).ti,ab,in. (79076)

66 (Gambia* or Guinea* or Guinea-Bissau* or Haiti* or Honduras* or Kenya* or Kiribati* or "North korea*" or "DPR Korea*" or "Korea* DPR" or "democratic people* republic of Korea*" or "Korea* democratic people* republic" or "DPRK").ti,ab,in. (113513)

67 (Liberia* or Madagasca* or Malawi* or Mali or Malinese or Mauritania* or Melanesia* or Micronesia* or Mozambique* or Myanmar* or Nepal* or Niger or Rwanda*).ti,ab,in. (87284)

68 ("Sao Tome*" or "São Tomé " or Principe* or Senegal* or "Sierra Leone" or "Solomon Islands" or Somalia* or Sudan or Tajikistan* or Tanzania* or "Timor-Leste" or Togo or Tuvalu or Uganda* or Vanuatu* or Yemen* or Zambia* or Zimbabw*).ti,ab,in. (105976)

69 or/1-68 [Lower income countries - DAC list] (2163643)

70 (Low-middle income* adj3 (countr* or nation* or economy or economies)).tw. (107)

71 (Lower-middle income* adj3 (countr* or nation* or economy or economies)).tw. (147)

72 (LMIC* adj3 (countr* or nation* or economy or economies)).tw. (468)

73 Armenia/ (3144)

74 Bhutan/ (1414)

75 Bolivia/ (7101)

76 west africa/ (121480)

77 Cape Verde/ (863)

78 Cameroon/ (12130)

79 Congo/ (3523)

80 Congo Democratic Republic/ (7465)

81 Cote d'Ivoire/ (9489)

82 Egypt/ (44251)

83 El Salvador/ (2303)

84 "republic of georgia"/ (3805)

85 Ghana/ (16361)

86 guatemala/ (6572)

87 guyana/ (3417)

88 Honduras/ (4120)

89 India/ (423204)

90 exp indonesia/ (37517)

91 Kenya/ (34382)

92 kosovo/ (533)

93 kyrgyzstan/ (2546)

94 Lesotho/ (1430)

95 Mauritania/ (1162)

96 Moldova/ (5787)

97 Mongolia/ (4449)

98 Morocco/ (13335)

99 Nicaragua/ (3690)

100 Nigeria/ (58243)

101 exp pakistan/ (42627)

102 papua new guinea/ (7683)

103 Paraguay/ (2978)

104 Philippines/ (27346)

105 samoa/ (1150)

106 Sri Lanka/ (17016)

107 Swaziland/ (2165)

108 Syria/ (5136)

109 tokelau/ (82)

110 Ukraine/ (27032)

111 Uzbekistan/ (8156)

112 vietnam/ (15391)

113 exp palestine/ (2263)

114 ("West Bank" or Gaza).ti,ab,in. (1471)

115 (Armenia* or Bolivia* or "Cabo Verde" or Cameroon* or Congo or "Cote D'Ivoire" or "Ivory coast" or Egypt* or "El Salvador" or Georgia* or Ghana* or Guatemala* or Guyana* or Honduras*).ti,ab,in. (223853)

116 (India or ((Indian or Indians) not "american indian*") or Indonesia* or Kenya* or Kosovo* or Kyrgyzstan* or Lesotho or Mauritania* or Mauritius or Moldova* or Mongolia* or Morocco* or Moroccan*).ti,ab,in. (856552)

117 (Nicaragua* or Nigeria* or Pakistan* or "Papua New Guinea*" or Paraguay or Philippines or Filipino* or Philipino* or Samoa* or "Sri Lanka*" or Swaziland* or Syria* or Ukrain* or Uzbekistan* or Vietnam*).ti,ab,in. (287811)

118 or/70-117 [LOWER MIDDLE Income countries LMIC World Bank and DAC list 2016] (1435655)

119 69 or 118 (2729577)

120 exp community development/ (18888)

121 social participation/ (9245)

122 community programmes/ (614)

123 (communit* adj3 (engage* or conversation* or action* or consult* or dialog*)).tw. (4598)

124 (communit* adj3 (particip* or involve* or action or empower* or collab* or led)).tw. (21313)

125 ((communit* or social) adj3 (mobilis* or mobiliz*)).tw. (1270)

126 or/120-125 [community engagement] (33248)

127 119 and 126 (20757)

128 exp bacterial diseases/ (233449)

129 exp viral diseases/ (248911)

130 exp protozoal infections/ (147167)

131 exp infectious diseases/ (33731)

132 exp diarrhoea/ (36692)

133 exp vector-borne diseases/ (168184)

134 exp diarrhoea/ (36692)

135 ((communicable or infectious or vector or mosquito* or insect* or water* or respiratory or gastrointestinal or sexual*) adj5 (disease* or infection* or transmission or transmit* or acquire*)).tw. (210746)

136 (HIV* or malaria* or TB or tuberculosis or polio* or diarrhea* or diarrhoea* or schistosomia*).tw. (255154)

137 (STD* or STI).tw. (10954)

138 exp immunization/ (86418)

139 (vaccinat* or immunis* or immuniz*).tw. (123318)

140 exp disease prevention/ or exp disease control/ (169730)

141 or/128-140 [communicable diseases and vaccination] (965436)

142 127 and 141 [community engagement in disease prevention] (2530)

143 ((systematic or metaanalys* or meta-analys* or rapid or evidence or qualitative or realist) adj2 (review or synthesis)).tw. (11892)

144 literature reviews/ or exp reviews/ (279357)

145 systematic reviews/ or meta-analysis/ (12243)

146 or/143-145 [review terms] (286558)

147 146 and 142 [Reviews of community engagement in disease prevention] (120)

148 limit 147 to yr="2007 -Current" (79)

## Database: Embase Classic+Embase <1947 to 2017 June 08>

**Search Strategy: EMB Community engagement disease LMIC Q3**

--------------------------------------------------------------------------------

1 Low income country/ (1083)

2 Developing country/ (87642)

3 (Low* income* adj3 (countr* or nation* or economy or economies)).tw. (6133)

4 (LIC* adj3 (countr* or nation* or economy or economies)).tw. (991)

5 ((Developing or underdeveloped or less-developed or "less* developed" or "third world") adj3 (countr* or nation* or economy or economies)).tw. (71373)

6 ((Underserved or "under served" or deprived or poor*) adj3 (countr* or nation* or economy or economies)).tw. (5714)

7 ((Developing or "less* developed" or less-developed or "under developed" or underdeveloped) adj2 world).tw. (9365)

8 ("Transitional country" or "transitional countries").tw. (191)

9 Afghanistan/ (4865)

10 Angola/ (1176)

11 Bangladesh/ (12587)

12 Benin/ (1960)

13 Bhutan/ (518)

14 Burkina Faso/ (3399)

15 Burundi/ (695)

16 Cambodia/ (3954)

17 Central African Republic/ (779)

18 Chad/ (878)

19 Comoros/ (270)

20 Democratic Republic of the Congo/ (3021)

21 Congo/ (3426)

22 Djibouti/ (293)

23 Equatorial Guinea/ (362)

24 Eritrea/ (435)

25 Ethiopia/ (11760)

26 Gambia/ (2471)

27 Guinea/ (2329)

28 Guinea-Bissau/ (927)

29 Haiti/ (3421)

30 Kenya/ (17147)

31 North Korea/ (416)

32 Lao People's Democratic Republic/ (1796)

33 Lesotho/ (517)

34 Liberia/ (1406)

35 Madagascar/ (3791)

36 Malawi/ (5393)

37 Mali/ (2849)

38 Mauritania/ (529)

39 Mozambique/ (2792)

40 Myanmar/ (2700)

41 Nepal/ (8720)

42 Niger/ (1944)

43 Rwanda/ (2610)

44 Senegal/ (6088)

45 Sierra Leone/ (1756)

46 Solomon Islands/ (420)

47 Somalia/ (1668)

48 South Sudan/ (87)

49 Sudan/ or Roraima/ (6459)

50 Tajikistan/ (792)

51 Tanzania/ (12254)

52 Togo/ (1185)

53 Uganda/ (14370)

54 Vanuatu/ (356)

55 Yemen/ (1697)

56 Zambia/ (5024)

57 Zimbabwe/ (5992)

58 (Afghanistan* or Angola* or "Atlantic Islands" or Bangladesh* or Benin* or Bhutan* or "Burkina Faso" or Burma or Burmese or Burundi* or Cambodia* or "Central African Republic" or Chad or Comoros or Congo or Djibouti*).ti,ab,in. (73270)

59 (Gambia* or Guinea* or Guinea-Bissau* or Haiti* or Honduras* or Kenya* or Kiribati* or "North korea*" or "DPR Korea*" or "Korea* DPR" or "democratic people* republic of Korea*" or "Korea* democratic people* republic" or "DPRK").ti,ab,in. (189746)

60 (Liberia* or Madagasca* or Malawi* or Mali or Malinese or Mauritania* or Melanesia* or Micronesia* or Mozambique* or Myanmar* or Nepal* or Niger or Rwanda*).ti,ab,in. (62355)

61 ("Sao Tome*" or "São Tomé " or Principe* or Senegal* or "Sierra Leone" or "Solomon Islands" or Somalia* or Sudan or Tajikistan* or Tanzania* or "Timor-Leste" or Togo or Tuvalu or Uganda* or Vanuatu* or Yemen* or Zambia* or Zimbabw*).ti,ab,in. (89476)

62 or/1-61 [Low income countries - DAC list 2016] (542254)

63 Armenia/ (1667)

64 Bolivia/ (3016)

65 Cabo Verde/ (0)

66 Cameroon/ (5732)

67 Congo/ (3426)

68 Cote d'Ivoire/ (2862)

69 Egypt/ (17988)

70 Cairo/ (34)

71 El Salvador/ (1505)

72 Georgia/ (502)

73 Ghana/ (8660)

74 Guatemala/ (3707)

75 Guyana/ (814)

76 Honduras/ (1567)

77 India/ or Assam/ or Andhra Pradesh/ or Assam/ or Bihar/ or Chhattisgarh/ or Chandigarh/ or Chhattisgarh/ or Goa/ or Gujarat/ or Jharkhand/ or Kashmir/ or Lakshadweep/ or Madhya Pradesh/ or Maharashtra/ or Manipur/ or Meghalaya/ or Mizoram/ or Nagaland/ or Odisha/ or Punjab/ or Tamil Nadu/ or Tripura/ or Uttar Pradesh/ or Uttarakhand/ or West Bengal/ (123579)

78 Indonesia/ or Irian Jaya/ or West Papua Province/ (12960)

79 Kosovo/ (283)

80 Kyrgyzstan/ (1350)

81 Micronesia/ (860)

82 Moldova/ (947)

83 Mongolia/ (2562)

84 Morocco/ (6457)

85 Nicaragua/ (1854)

86 Nigeria/ (32661)

87 Pakistan/ or Azad Jammu/ or Kashmir, Rajasthan/ (21341)

88 Papua New Guinea/ (6103)

89 Paraguay/ (1120)

90 Philippines/ (9833)

91 Philippines/ or Manila/ (9836)

92 Samoa/ (534)

93 Sri Lanka/ (6933)

94 Swaziland/ (719)

95 Syrian Arab Republic/ or Syria/ (1937)

96 Tokelau/ (46)

97 Ukraine/ (15135)

98 Uzbekistan/ (1958)

99 Vietnam/ (12677)

100 West Bank/ (7)

101 Gaza strip/ (6)

102 (Armenia* or Bolivia* or "Cabo Verde" or Cameroon* or Congo or "Cote D'Ivoire" or "Ivory coast" or Egypt* or "El Salvador" or Georgia* or Ghana* or Guatemala* or Guyana* or Honduras*).ti,ab,in. (271536)

103 (India or ((Indian or Indians) not "american indian*") or Indonesia* or Kenya* or Kosovo* or Kyrgyzstan* or Lesotho or Mauritania* or Mauritius or Moldova* or Mongolia* or Morocco* or Moroccan*).ti,ab,in. (791277)

104 (Nicaragua* or Nigeria* or Pakistan* or "Papua New Guinea*" or Paraguay or Philippines or Filipino* or Samoa* or "Sri Lanka*" or Swaziland* or Syria* or Ukrain* or Uzbekistan* or Vietnam*).ti,ab,in. (247241)

105 (West bank or Gaza Strip).ti,ab,in. (1779)

106 or/63-105 [Lower Middle Income Countries and Territories - DAC List of ODA Recipients 2016] (1344749)

107 or/62,106 [Low or Lower Middle Income Countries - DAC list of ODA recipients 2016] (1756218)

108 community participation/ (306)

109 *community/ (16036)

110 *community care/ (21567)

111 participatory research/ (3585)

112 (communit* adj3 (engage* or conversation* or action* or consult* or dialog*)).tw. (6893)

113 (communit* adj3 (particip* or involve* or action or empower* or collab* or led)).tw. (27014)

114 ((communit* or social) adj3 (mobilis* or mobiliz*)).tw. (1848)

115 or/108-114 [community engagement] (68171)

116 107 and 115 (8892)

117 infection/ or exp bacterial infection/ or exp sexually transmitted disease/ or exp virus infection/ or water borne disease/ or exp zoonosis/ (2189491)

118 exp mycosis/ (181748)

119 communicable disease/ or community acquired infection/ or exp hemorrhagic fever/ or exp parasitosis/ or exp respiratory tract infection/ (759921)

120 exp tropical disease/ (163200)

121 exp diarrhea/ (224910)

122 ((communicable or infectious or vector or mosquito* or insect* or water* or respiratory or gastrointestinal or sexual*) adj5 (disease* or infection* or transmission or transmit* or acquire*)).tw. (332064)

123 (HIV* or malaria* or TB or tuberculosis or polio* or diarrhea* or diarrhoea* or schistosomia*).tw. (814722)

124 (STD* or STI).tw. (29571)

125 exp immunization/ (275897)

126 (vaccinat* or immunis* or immuniz*).tw. (287425)

127 exp disease control/ (184310)

128 or/117-127 [communicable diseases and vaccination] (3396714)

129 116 and 128 [Community engagement in disease prevention - LMIC] (3530)

130 limit 129 to (meta analysis or "systematic review") (44)

131 ((systematic or metaanalys* or meta-analys* or rapid or evidence or qualitative or realist) adj2 (review or synthesis)).tw. (144007)

132 129 and 131 (44)

133 130 or 132 (58)

134 limit 133 to yr="2007 -Current" (57)

## Database: Global Health <1910 to 2017 Week 22>

**Search Strategy:GLO Community Engagment Disease LMIC Q3**

--------------------------------------------------------------------------------

1 Developing Countries/ (792651)

2 (Low* income* adj3 (countr* or nation* or economy or economies)).tw. (3040)

3 (LIC* adj3 (countr* or nation* or economy or economies)).tw. (180)

4 ((Developing or "under developed" or underdeveloped or less-developed or "less* developed" or "third world") adj3 (countr* or nation* or economy or economies)).tw. (801159)

5 ((Underserved or "under served" or deprived or poor*) adj3 (countr* or nation* or economy or economies)).tw. (2783)

6 ((Developing or "less* developed" or less-developed or "under developed" or underdeveloped) adj2 world).tw. (3757)

7 ("Transitional country" or "transitional countries" or "transitioning country" or "transitioning countries").tw. (101)

8 Afghanistan/ (1629)

9 Central Africa/ (18291)

10 East Africa.gl. (3950)

11 Angola/ (1253)

12 "Sao Tome and Principe"/ (209)

13 Bangladesh/ (9476)

14 Benin/ (2132)

15 Burkina Faso/ (4137)

16 Burundi/ (608)

17 Cambodia/ (2448)

18 Central African Republic/ (995)

19 Chad/ (1094)

20 Comoros/ (274)

21 Djibouti/ (308)

22 Equatorial Guinea/ (367)

23 Eritrea/ (586)

24 Ethiopia/ (9671)

25 Gambia/ (2737)

26 Guinea/ (995)

27 Guinea-Bissau/ (901)

28 Haiti/ (1811)

29 Kenya/ (14969)

30 Korea Democratic People's Republic/ (260)

31 Laos/ (1172)

32 Lesotho/ (521)

33 Liberia/ (1425)

34 Madagascar/ (3489)

35 Malawi/ (4954)

36 Mali/ (2731)

37 Mauritania/ (469)

38 Melanesia/ [MeSH term for Solomon Islands] (7219)

39 Micronesia/ [MeSH term for Kiribati and Tuvalu] (1062)

40 "Federated States of Micronesia"/ (149)

41 Kiribati/ (133)

42 Solomon Islands/ (753)

43 Tuvalu/ (98)

44 Mozambique/ (2521)

45 Myanmar/ (3238)

46 Nepal/ (5177)

47 Niger/ (1172)

48 Rwanda/ (1734)

49 Senegal/ (5825)

50 Sierra Leone/ (1807)

51 Somalia/ (1498)

52 South Sudan/ (187)

53 Sudan/ (6479)

54 Tajikistan/ (613)

55 Tanzania/ (11310)

56 Togo/ (1459)

57 Uganda/ (11065)

58 Vanuatu/ (565)

59 Yemen/ (1477)

60 Zambia/ (4310)

61 Zimbabwe/ (5807)

62 (Afghan* or Angola* or "Atlantic Islands" or Bangladesh* or Benin* or Bhutan* or "Burkina Faso" or Burma or Burmese or Burundi* or Cambodia* or "Central African Republic" or Chad or Comoros or Djibouti*).ti,ab,in. (29883)

63 (Gambia* or Guinea* or Guinea-Bissau* or Haiti* or Honduras* or Kenya* or Kiribati* or "North korea*" or "DPR Korea*" or "Korea* DPR" or "democratic people* republic of Korea*" or "Korea* democratic people* republic" or "DPRK").ti,ab,in. (71848)

64 (Lao or Laos or Lesotho* or Liberia* or Madagasca* or Malawi* or Mali or Malinese or Mauritania* or Melanesia* or Micronesia* or Mozambique* or Myanmar* or Nepal* or Niger or Rwanda*).ti,ab,in. (37880)

65 (Senegal* or "Sierra Leone" or "Solomon Islands" or Somalia* or Sudan* or Tajikistan* or Tanzania* or Timor* or Togo or Uganda* or Vanuatu* or Yemen* or Zambia* or Zimbabw*).ti,ab,in. (50321)

66 or/1-65 [LOWER income countries - DAC and World Bank list] (861186)

67 (Low-middle income* adj3 (countr* or nation* or economy or economies)).tw. (180)

68 (Lower-middle income* adj3 (countr* or nation* or economy or economies)).tw. (209)

69 (LMIC* adj3 (countr* or nation* or economy or economies)).tw. (822)

70 Armenia/ (607)

71 Bhutan/ (349)

72 Bolivia/ (2394)

73 West Africa/ (63764)

74 Cape Verde/ (283)

75 Cameroon/ (6301)

76 Congo/ (1975)

77 "Democratic Republic of the Congo"/ (5763)

78 Cote d'Ivoire/ (4123)

79 Egypt/ (16301)

80 El Salvador/ (1222)

81 "Republic of Georgia"/ (702)

82 Ghana/ (8003)

83 Guatemala/ (3286)

84 Guyana/ (1076)

85 Honduras/ (1218)

86 India/ (102829)

87 Indonesia/ (10848)

88 Kenya/ (14969)

89 Kosovo/ (261)

90 Kyrgyzstan/ (563)

91 Lesotho/ (521)

92 Mauritania/ (469)

93 Moldova/ (572)

94 Mongolia/ (1085)

95 Morocco/ (5580)

96 Nicaragua/ (1162)

97 Nigeria/ (28608)

98 Pakistan/ (13727)

99 Papua New Guinea/ (3165)

100 Paraguay/ (1095)

101 Philippines/ (7584)

102 Samoa/ (410)

103 Sri Lanka/ (5692)

104 Swaziland/ (659)

105 Syria/ (1233)

106 Tokelau.ti,ab,in. (127)

107 Ukraine/ (2979)

108 Uzbekistan/ (1579)

109 Vietnam/ (7681)

110 ("West Bank" or Gaza).ti,ab,in. (771)

111 (Armenia* or Bolivia* or "Cabo Verde" or Cameroon* or Congo or "Cote D'Ivoire" or "Ivory coast" or Egypt* or "El Salvador" or Georgia* or Ghana* or Guatemala* or Guyana* or Honduras*).ti,ab,in. (82661)

112 (India or ((Indian or Indians) not "american indian*") or Indonesia* or Kenya* or Kosovo* or Kyrgyzstan* or Lesotho or Mauritania* or Mauritius or Moldova* or Mongolia* or Morocco* or Moroccan*).ti,ab,in. (231105)

113 (Nicaragua* or Nigeria* or Pakistan* or "Papua New Guinea*" or Paraguay or Philippines or Filipino* or Samoa* or "Sri Lanka*" or Swaziland* or Syria* or Ukrain* or Uzbekistan* or Vietnam*).ti,ab,in. (95180)

114 or/67-113 [LOWER-MIDDLE income countries LMIC- World Bank and DAC list 2016] (440975)

115 66 or 114 (1014154)

116 exp community development/ (4292)

117 social participation/ (1351)

118 community programmes/ (1008)

119 (communit* adj3 (engage* or conversation* or action* or consult* or dialog*)).tw. (2627)

120 (communit* adj3 (particip* or involve* or action or empower* or collab* or led)).tw. (10242)

121 ((communit* or social) adj3 (mobilis* or mobiliz*)).tw. (1099)

122 or/116-121 [community engagement] (13807)

123 115 and 122 (6379)

124 exp infectious diseases/ (47644)

125 exp bacterial diseases/ (328266)

126 exp viral diseases/ (350101)

127 waterborne diseases/ (4078)

128 exp parasitoses/ (251009)

129 exp vector-borne diseases/ (203369)

130 exp diarrhoea/ (37420)

131 ((communicable or infectious or vector or mosquito* or insect* or water* or respiratory or gastrointestinal or sexual*) adj5 (disease* or infection* or transmission or transmit* or acquire*)).tw. (234789)

132 (HIV* or malaria* or TB or tuberculosis or polio* or diarrhea* or diarrhoea* or schistosomia*).tw. (404958)

133 (STD* or STI).tw. (31418)

134 exp immunization/ (90045)

135 immunization programmes/ (3970)

136 exp disease control/ or exp disease prevention/ (127139)

137 or/124-136 [communicable diseases and vaccination] (1104506)

138 123 and 137 [Community engagement in disease prevention - LMIC] (2920)

139 systematic reviews/ or meta-analysis/ (26039)

140 literature reviews/ (13116)

141 ((systematic or metaanalys* or meta-analys* or rapid or evidence or qualitative or realist) adj2 (review or synthesis)).tw. (21205)

142 or/139-141 [review terms] (41386)

143 142 and 138 (56)

144 limit 143 to yr="2007 -Current" (55)

## Ovid MEDLINE(R) Epub Ahead of Print, In-Process & Other Non-Indexed Citations, Ovid MEDLINE(R) Daily and Ovid MEDLINE(R) <1946 to Present>

**Search Strategy: MED Community Engagement disease LMIC Q3**

--------------------------------------------------------------------------------

1 Developing country/ (70242)

2 (Low* income* adj3 (countr* or nation* or economy or economies)).tw. (5259)

3 (LIC* adj3 (countr* or nation* or economy or economies)).tw. (802)

4 ((Developing or "under developed" or underdeveloped or less-developed or "less* developed" or "third world") adj3 (countr* or nation* or economy or economies)).tw. (58662)

5 ((Underserved or "under served" or deprived or poor*) adj3 (countr* or nation* or economy or economies)).tw. (4876)

6 ((Developing or "less* developed" or less-developed or "under developed" or underdeveloped) adj2 world).tw. (7881)

7 ("Transitional country" or "transitional countries" or "transitioning country" or "transitioning countries").tw. (157)

8 Afghanistan/ (2785)

9 Africa, Central/ (1162)

10 Africa, Eastern/ (3809)

11 Angola/ (810)

12 Atlantic Islands/ [MeSH term for Sao Tome and Principe] (721)

13 Bangladesh/ (8737)

14 Benin/ (1291)

15 Burkina Faso/ (2757)

16 Burundi/ (586)

17 Cambodia/ (2803)

18 Central African Republic/ (712)

19 Chad/ (620)

20 Comoros/ (254)

21 Djibouti/ (202)

22 Equatorial Guinea/ (214)

23 Eritrea/ (261)

24 Ethiopia/ (9191)

25 Gambia/ (2314)

26 Guinea/ (835)

27 Guinea-Bissau/ (855)

28 Haiti/ (2790)

29 Kenya/ (13505)

30 "Democratic People's Republic of Korea"/ (164)

31 Laos/ (1592)

32 Lesotho/ (349)

33 Liberia/ (983)

34 Madagascar/ (2918)

35 Malawi/ (4119)

36 Mali/ (2073)

37 Mauritania/ (389)

38 Melanesia/ [MeSH term for Solomon Islands] (971)

39 Micronesia/ [MeSH term for Kiribati and Tuvalu] (1070)

40 Mozambique/ (1869)

41 Myanmar/ (1742)

42 Nepal/ (6421)

43 Niger/ (1040)

44 Rwanda/ (1872)

45 Senegal/ (5209)

46 Sierra Leone/ (1187)

47 Somalia/ (1335)

48 South Sudan/ (40)

49 Sudan/ (4378)

50 Tajikistan/ (679)

51 Tanzania/ (9521)

52 Timor-Leste/ (126)

53 Togo/ (971)

54 Uganda/ (9929)

55 Vanuatu/ (310)

56 Yemen/ (1225)

57 Zambia/ (3797)

58 Zimbabwe/ (5253)

59 (Afghan* or Angola* or "Atlantic Islands" or Bangladesh* or Benin* or Bhutan* or "Burkina Faso" or Burma or Burmese or Burundi* or Cambodia* or "Central African Republic" or Chad or Comoros or Djibouti*).ti,ab,in. (46834)

60 (Gambia* or Guinea* or Guinea-Bissau* or Haiti* or Honduras* or Kenya* or Kiribati* or "North korea*" or "DPR Korea*" or "Korea* DPR" or "democratic people* republic of Korea*" or "Korea* democratic people* republic" or "DPRK").ti,ab,in. (140980)

61 (Lao or Laos or Lesotho* or Liberia* or Madagasca* or Malawi* or Mali or Malinese or Mauritania* or Melanesia* or Micronesia* or Mozambique* or Myanmar* or Nepal* or Niger or Rwanda*).ti,ab,in. (52398)

62 (Senegal* or "Sierra Leone" or "Solomon Islands" or Somalia* or Sudan* or Tajikistan* or Tanzania* or Timor* or Togo or Uganda* or Vanuatu* or Yemen* or Zambia* or Zimbabw*).ti,ab,in. (63012)

63 or/1-62 [LOWER income countries - DAC list] (417733)

64 (Low-middle income* adj3 (countr* or nation* or economy or economies)).tw. (395)

65 (Lower-middle income* adj3 (countr* or nation* or economy or economies)).tw. (396)

66 (LMIC* adj3 (countr* or nation* or economy or economies)).tw. (1840)

67 Armenia/ (1288)

68 Bhutan/ (296)

69 Bolivia/ (2222)

70 Africa, Western/ (5300)

71 Cape Verde/ (141)

72 Cameroon/ (4604)

73 Congo/ (1617)

74 "Democratic Republic of the Congo"/ (3564)

75 Cote d'Ivoire/ (2799)

76 Egypt/ (13680)

77 El Salvador/ (801)

78 "Georgia (Republic)"/ (1552)

79 Ghana/ (6286)

80 Guatemala/ (2673)

81 Guyana/ (610)

82 Honduras/ (993)

83 India/ (90816)

84 Indonesia/ (8636)

85 Kenya/ (13505)

86 Kosovo/ (99)

87 Kyrgyzstan/ (1217)

88 Lesotho/ (349)

89 Mauritania/ (389)

90 Moldova/ (637)

91 Mongolia/ (1480)

92 Morocco/ (4869)

93 Nicaragua/ (1307)

94 Nigeria/ (25583)

95 Pakistan/ (14506)

96 Papua New Guinea/ (3183)

97 Paraguay/ (678)

98 Philippines/ (7529)

99 Samoa/ (282)

100 Sri Lanka/ (5097)

101 Swaziland/ (434)

102 Syria/ (1309)

103 Tokelau.ti,ab,in. (92)

104 Ukraine/ (15172)

105 Uzbekistan/ (1870)

106 Vietnam/ (10426)

107 ("West Bank" or Gaza).ti,ab,in. (1817)

108 (Armenia* or Bolivia* or "Cabo Verde" or Cameroon* or Congo or "Cote D'Ivoire" or "Ivory coast" or Egypt* or "El Salvador" or Georgia* or Ghana* or Guatemala* or Guyana* or Honduras*).ti,ab,in. (207975)

109 (India or ((Indian or Indians) not "american indian*") or Indonesia* or Kenya* or Kosovo* or Kyrgyzstan* or Lesotho or Mauritania* or Mauritius or Moldova* or Mongolia* or Morocco* or Moroccan*).ti,ab,in. (486121)

110 (Nicaragua* or Nigeria* or Pakistan* or "Papua New Guinea*" or Paraguay or Philippines or Filipino* or Samoa* or "Sri Lanka*" or Swaziland* or Syria* or Ukrain* or Uzbekistan* or Vietnam*).ti,ab,in. (148456)

111 or/64-110 [LOWER-MIDDLE income countries LMIC- World Bank and DAC list 2016] (897814)

112 63 or 111 (1225051)

113 Community Participation/ (15898)

114 Community-Based Participatory Research/ (3297)

115 Community-Institutional Relations/ (10389)

116 Community Networks/ (6438)

117 (communit* adj3 (engage* or conversation* or action* or consult* or dialog*)).tw. (5663)

118 (communit* adj3 (particip* or involve* or action or empower* or collab* or led)).tw. (21723)

119 ((communit* or social) adj3 (mobilis* or mobiliz*)).tw. (1687)

120 or/113-119 [Community engagement] (54662)

121 112 and 120 (7064)

122 exp "bacterial infections and mycoses"/ or exp mycoses/ or exp zoonoses/ or exp virus diseases/ or exp parasitic diseases/ (2286385)

123 Diarrhea/ (44213)

124 ((communicable or infectious or vector or mosquito* or insect* or water* or respiratory or gastrointestinal or sexual*) adj5 (disease* or infection* or transmission or transmit* or acquire*)).tw. (245100)

125 (HIV* or malaria* or TB or tuberculosis or polio* or diarrhea* or diarrhoea* or schistosomia*).tw. (647536)

126 (STD* or STI).tw. (19948)

127 exp Immunization/ (159950)

128 (vaccinat* or immunis* or immuniz*).tw. (229994)

129 exp Communicable Disease Control/ (302169)

130 or/122-129 [communicable diseases and vaccination] (2893892)

131 121 and 130 [community engagement in disease prevention] (2703)

132 limit 131 to (meta analysis or systematic reviews) (95)

133 ((systematic or metaanalys* or meta-analys* or rapid or evidence or qualitative or realist) adj2 (review or synthesis)).tw. (121191)

134 131 and 133 (33)

135 132 or 134 [Community engagement disease and immunisation] (100)

136 limit 135 to yr="2007 -Current" (91)

## Cochrane Library (43)

Search Name: Community Engagement Disease LMIC Q3

Date Run: 09/06/17 15:48:23.270

Description: 09-06-17

### Database of Abstracts of Reviews of Effect : Issue 2 of 4, April 2015 (1)

ID Search Hits

#1 MeSH descriptor: [Developing Countries] explode all trees 1011

#2 ((low* next income) near/3 (countr* or nation* or economy or economies)):ti,ab,kw 416

#3 (LIC* near/3 (countr* or nation* or economy or economies)):ti,ab,kw 43

#4 ((developing or "under developed" or underdeveloped or less-developed or "less developed" or "third world") near/3 (countr* or nation* or economy or economies)):ti,ab,kw 3078

#5 ((Underserved or "under served" or deprived or poor*) near/3 (countr* or nation* or economy or economies)):ti,ab,kw 158

#6 ((Developing or "less* developed" or less-developed or "under developed" or underdeveloped) near/2 world):ti,ab,kw 221

#7 ("Transitional country" or "transitional countries" or "transitioning country" or "transitioning countries"):ti,ab,kw 3

#8 MeSH descriptor: [Afghanistan] explode all trees 36

#9 MeSH descriptor: [Africa, Central] explode all trees 247

#10 MeSH descriptor: [Africa, Eastern] explode all trees 1820

#11 MeSH descriptor: [Angola] explode all trees 11

#12 MeSH descriptor: [Atlantic Islands] explode all trees 2

#13 MeSH descriptor: [Bangladesh] explode all trees 482

#14 MeSH descriptor: [Benin] explode all trees 46

#15 MeSH descriptor: [Burkina Faso] explode all trees 111

#16 MeSH descriptor: [Burundi] explode all trees 10

#17 MeSH descriptor: [Cambodia] explode all trees 82

#18 MeSH descriptor: [Central African Republic] explode all trees 12

#19 MeSH descriptor: [Chad] explode all trees 7

#20 MeSH descriptor: [Comoros] explode all trees 1

#21 MeSH descriptor: [Djibouti] explode all trees 1

#22 MeSH descriptor: [Equatorial Guinea] explode all trees 2

#23 MeSH descriptor: [Eritrea] explode all trees 2

#24 MeSH descriptor: [Ethiopia] explode all trees 140

#25 MeSH descriptor: [Gambia] explode all trees 209

#26 MeSH descriptor: [Guinea] explode all trees 8

#27 MeSH descriptor: [Guinea-Bissau] explode all trees 81

#28 MeSH descriptor: [Haiti] explode all trees 51

#29 MeSH descriptor: [Kenya] explode all trees 553

#30 MeSH descriptor: [Democratic People's Republic of Korea] explode all trees 5

#31 MeSH descriptor: [Laos] explode all trees 22

#32 MeSH descriptor: [Lesotho] explode all trees 4

#33 MeSH descriptor: [Liberia] explode all trees 16

#34 MeSH descriptor: [Madagascar] explode all trees 36

#35 MeSH descriptor: [Malawi] explode all trees 243

#36 MeSH descriptor: [Mali] explode all trees 75

#37 MeSH descriptor: [Mauritania] explode all trees 4

#38 MeSH descriptor: [Melanesia] explode all trees 75

#39 MeSH descriptor: [Micronesia] explode all trees 4

#40 MeSH descriptor: [Mozambique] explode all trees 67

#41 MeSH descriptor: [Myanmar] explode all trees 55

#42 MeSH descriptor: [Nepal] explode all trees 221

#43 MeSH descriptor: [Niger] explode all trees 33

#44 MeSH descriptor: [Rwanda] explode all trees 41

#45 MeSH descriptor: [Senegal] explode all trees 82

#46 MeSH descriptor: [Sierra Leone] explode all trees 28

#47 MeSH descriptor: [Somalia] explode all trees 11

#48 MeSH descriptor: [South Sudan] explode all trees 0

#49 MeSH descriptor: [Sudan] explode all trees 81

#50 MeSH descriptor: [Tajikistan] explode all trees 2

#51 MeSH descriptor: [Tanzania] explode all trees 469

#52 MeSH descriptor: [Timor-Leste] explode all trees 1

#53 MeSH descriptor: [Togo] explode all trees 14

#54 MeSH descriptor: [Uganda] explode all trees 529

#55 MeSH descriptor: [Vanuatu] explode all trees 2

#56 MeSH descriptor: [Yemen] explode all trees 9

#57 MeSH descriptor: [Zambia] explode all trees 217

#58 MeSH descriptor: [Zimbabwe] explode all trees 158

#59 (Afghan* or Angola* or "Atlantic Islands" or Bangladesh* or Benin* or Bhutan* or "Burkina Faso" or Burma or Burmese or Burundi* or Cambodia* or "Central African Republic" or Chad or Comoros or Djibouti*):ti,ab,kw 1927

#60 (Gambia* or Guinea* or Guinea-Bissau* or Haiti* or Honduras* or Kenya* or Kiribati* or "North korea*" or "DPR Korea*" or "Korea* DPR" or "democratic people* republic of Korea*" or "Korea* democratic people* republic" or "DPRK"):ti,ab,kw 2372

#61 (Lao or Laos or Lesotho* or Liberia* or Madagasca* or Malawi* or Mali or Malinese or Mauritania* or Melanesia* or Micronesia* or Mozambique* or Myanmar* or Nepal* or Niger or Rwanda*):ti,ab,kw 2103

#62 (Senegal* or "Sierra Leone" or "Solomon Islands" or Somalia* or Sudan* or Tajikistan* or Tanzania* or Timor* or Togo or Uganda* or Vanuatu* or Yemen* or Zambia* or Zimbabw*):ti,ab,kw 3271

#63 {or #1-#62} 12217

#64 (Low-middle income* near/3 (countr* or nation* or economy or economies)):ti,ab,kw 46

#65 (Lower-middle income* near/3 (countr* or nation* or economy or economies)):ti,ab,kw 23

#66 (LMIC* near/3 (countr* or nation* or economy or economies)):ti,ab,kw 98

#67 MeSH descriptor: [Armenia] explode all trees 10

#68 MeSH descriptor: [Bhutan] explode all trees 1

#69 MeSH descriptor: [Bolivia] explode all trees 37

#70 MeSH descriptor: [Africa, Western] this term only 29

#71 MeSH descriptor: [Cape Verde] explode all trees 0

#72 MeSH descriptor: [Cameroon] explode all trees 95

#73 MeSH descriptor: [Congo] explode all trees 14

#74 MeSH descriptor: [Democratic Republic of the Congo] explode all trees 72

#75 MeSH descriptor: [Cote d'Ivoire] explode all trees 76

#76 MeSH descriptor: [Egypt] explode all trees 291

#77 MeSH descriptor: [El Salvador] explode all trees 5

#78 MeSH descriptor: [Georgia (Republic)] explode all trees 14

#79 MeSH descriptor: [Ghana] explode all trees 228

#80 MeSH descriptor: [Guatemala] explode all trees 110

#81 MeSH descriptor: [Guyana] explode all trees 3

#82 MeSH descriptor: [Honduras] explode all trees 35

#83 MeSH descriptor: [India] explode all trees 1757

#84 MeSH descriptor: [Indonesia] explode all trees 268

#85 MeSH descriptor: [Kenya] explode all trees 553

#86 MeSH descriptor: [Kosovo] explode all trees 2

#87 MeSH descriptor: [Kyrgyzstan] explode all trees 6

#88 MeSH descriptor: [Lesotho] explode all trees 4

#89 MeSH descriptor: [Mauritania] explode all trees 4

#90 MeSH descriptor: [Moldova] explode all trees 1

#91 MeSH descriptor: [Mongolia] explode all trees 18

#92 MeSH descriptor: [Morocco] explode all trees 34

#93 MeSH descriptor: [Nicaragua] explode all trees 27

#94 MeSH descriptor: [Nigeria] explode all trees 494

#95 MeSH descriptor: [Pakistan] explode all trees 339

#96 MeSH descriptor: [Papua New Guinea] explode all trees 56

#97 MeSH descriptor: [Paraguay] explode all trees 5

#98 MeSH descriptor: [Philippines] explode all trees 139

#99 MeSH descriptor: [Samoa] explode all trees 6

#100 MeSH descriptor: [Sri Lanka] explode all trees 84

#101 MeSH descriptor: [Swaziland] explode all trees 9

#102 MeSH descriptor: [Syria] explode all trees 16

#103 Tokelau:ti,ab,kw 1

#104 MeSH descriptor: [Ukraine] explode all trees 48

#105 MeSH descriptor: [Uzbekistan] explode all trees 9

#106 MeSH descriptor: [Vietnam] explode all trees 288

#107 ("West Bank" or Gaza):ti,ab,kw 35

#108 (Armenia* or Bolivia* or "Cabo Verde" or Cameroon* or Congo or "Cote D'Ivoire" or "Ivory coast" or Egypt* or "El Salvador" or Georgia* or Ghana* or Guatemala* or Guyana* or Honduras*):ti,ab,kw 3951

#109 (India or ((Indian or Indians) not "american indian*") or Indonesia* or Kenya* or Kosovo* or Kyrgyzstan* or Lesotho or Mauritania* or Mauritius or Moldova* or Mongolia* or Morocco* or Moroccan*):ti,ab,kw 8265

#110 (Nicaragua* or Nigeria* or Pakistan* or "Papua New Guinea*" or Paraguay or Philippines or Filipino* or Samoa* or "Sri Lanka*" or Swaziland* or Syria* or Ukrain* or Uzbekistan* or Vietnam*):ti,ab,kw 3798

#111 {or #64-#110} 15642

#112 #63 or #111 24933

#113 MeSH descriptor: [Community Participation] explode all trees 1328

#114 MeSH descriptor: [Community-Based Participatory Research] explode all trees 180

#115 MeSH descriptor: [Community-Institutional Relations] explode all trees 199

#116 MeSH descriptor: [Community Networks] explode all trees 163

#117 (communit* near/3 (engage* or conversation* or action* or consult* or dialog*)):ti,ab 360

#118 (communit* near/3 (particip* or involve* or action or empower* or collab* or led)):ti,ab 2203

#119 ((communit* or social) near/3 (mobilis* or mobiliz*)):ti,ab 136

#120 {or #113-#119} 4076

#121 #112 and #119 84

#122 MeSH descriptor: [Bacterial Infections and Mycoses] explode all trees 31623

#123 MeSH descriptor: [Virus Diseases] explode all trees 22261

#124 MeSH descriptor: [Parasitic Diseases] explode all trees 5752

#125 MeSH descriptor: [Diarrhea] explode all trees 2815

#126 ((communicable or infectious or vector or mosquito* or insect* or water* or respiratory or gastrointestinal or sexual*) near/5 (disease* or infection* or transmission or transmit* or acquire*)):ti,ab 10956

#127 (HIV* or malaria* or TB or tuberculosis or polio* or diarrhea* or diarrhoea* or schistosomia*):ti,ab 33646

#128 (STD* or STI):ti,ab 1474

#129 MeSH descriptor: [Immunization] explode all trees 4801

#130 (vaccinat* or immunis* or immuniz*):ti,ab 10855

#131 MeSH descriptor: [Communicable Disease Control] explode all trees 5163

#132 {or #122-#131} 88501

#133 #121 and #132 Publication Year from 2007 to 2017 43
