# Supplementary material for: Community engagement interventions for communicable disease control in low- and lower- middle-income countries: evidence from a review of systematic reviews
Source: Int J Equity Health. 2020 Apr 6;19:51. doi: 10.1186/s12939-020-01169-5 (PMC7137248; doi:10.1186/s12939-020-01169-5)
Supplement: Supplementary file 2 — Additional file 2: Table S1. Characteristics of Included Systematic Reviews. [file 12939_2020_1169_MOESM2_ESM.docx]

**Table 1: Characteristics of Included Systematic Reviews**

| **Author, year** | **Health topic** | **Number of primary studies meeting review of reviews criteria*** | **Design of primary studies** | **Countries included** | **Target populations of included studies** |
| --- | --- | --- | --- | --- | --- |
| Skevington et al., 2013 [20] | HIV | 5 | Longitudinal survey (4)  (one with no baseline) and  pilot structured evaluation with mixed methods (1). | The Gambia (2), India (1), Ethiopia (1), Angola/ Tanzania/ Uganda (1) | Men and women |
| Cornish et al., 2014, [7] | HIV | 10 studies from 18 publications. | RCT (4), Cohort analytic (3), Case control (2), Cohort (1). | Uganda (1), Tanzania (1), Zimbabwe (2), Tanzania/ Zimbawbe/ Thailand (1)  India (5). | Varied: Adults, young people, married men and those with high risk behaviours: female sex workers (FSW), men who have sex with men (MSM), injecting drug users, truck drivers. |
| Kerrigan et al., 2013 [8] | HIV | 7 | Randomized group trial (1) , cross sectional study (6). | India (7) | Sex workers. |
| Kerrigan et al., 2015 [9] | HIV | 17 | Group randomised trial (1), serial cross-sectional study (9), cross-sectional study (7). | India (17) | Sex-workers. |
| Nachega et al., 2016, [21] | HIV | 11 | RCT (7), cluster RCT (2), cohort (1), prospective cohort (1). | Uganda (5), Nigeria (2), Kenya (1), Rwanda (1), Tanzania and Zambia (1),  Botswana/Brazil/Haiti/Peru/ South Africa/Uganda/ Zambia/ Zimbabwe (1). | People living with HIV and AIDS. |
| Medley et al., 2009, [22] | HIV | 15 | Randomised trial (1), before/after with no comparison group (3),  prospective cohort (1), cross sectional (7), time series (3). | Kenya (3), Phillipines (2), Indonesia (2), Zambia (1), Cameroon (1), Ghana (1), Zimbabwe (1), Senegal (1), Mozambique (1), Tanzania (1), Malawi (1). | Adolescents (some were >18 years), female commercial sex workers, truck drivers/ transport workers, adult heterosexual men and women, prisoners. |
| Atkinson et al., 2011, [25] | Malaria | 40 | Qualitative (2), effectiveness study (5), RCT (1), analytical (2), case reports (30). | Cameroon (4), Zimbabwe (1), Nigeria (2), Zaire (1), Tanzania (1), India (2), Cambodia (1), Uganda (1), Nicaragua (2), Vanuatu (1), Indonesia (2), Guatemala (1), Honduras (1), Papua New Guinea (1), Carribean (1), Tanzania (3), India (2), India + Pakistan (1), Loa (1), Zimbabwe (1), Kenya (2), Ethiopia (1), Nigeria (1)  Global (2), Bolivia (1), Morocco (1), Mali (1), Sri Lanka (1). | Men and women, adolescents in one study, committee members or women with children < 5 years, patients presenting with fever in the community. |
| Okwundu et al., 2013, [26] | Malaria | 10 | Cluster RCTs (7), controlled before-and-after studies (3). | Tanzania (2), Kenya (2), Uganda (2), Democratic Republic of Congo (1), Ethiopia (1), Burkina Faso (1), Zambia (1). | Community volunteers , women (leaders/ mothers/ caregivers), retail outlet staff, community health workers. |
| Musa et al., 2014, [27] | TB | 10 | Cluster RCT (3), RCT (2), non-randomised controlled trial (1), cohort study (3), community randomised trial (1). | Ethiopia (3), Tanzania (2), Swaziland (1), Cambodia (1), Namibia (1), Uganda (1), Zimbabwe (1). | Patients with tuberculosis. |
| Farnsworth et al., 2014, [2]. | Child and maternal health | 24 studies from 31 publications. | Cluster RCT (8), RCT (4), qualitative process evaluation of RCT (2),  quasi-experimental study with control (1), pre-post studies (5), prospective observational cohort (1), case control study (1), follow up survey (1), secondary analysis of survey data (1), project evaluation (2), coverage estimation study (1). | India (9), Pakistan (1), Kenya (2), Zambia (2), Bangladesh (5) Honduras/Peru (1), Benin (1), Vietnam (2), Ghana (1), Malawi (2), Nepal (3), Bolivia (1), Tanzania (1). | Pregnant women, women of reproductive age, families or households. |
| Prost et al., 2014 [23] | Child and maternal health | 7 | Cluster RCTs (7) | Nepal (1), Bangladesh (2), Malawi (2), India (2) | Women of reproductive age, mostly 15-49 years. |
| Gilmore and McAuliffe, 2013, [24] | Child and maternal health | 14 | Before/after studies (4) , RCT (3), cluster RCT (5), cross sectional nested in RCT (1), case series (1). | Bangladesh (5), India (3), Pakistan (2), Nigeria (1), Uganda (1), Philippines (1), Burkina Faso (1). | Pregnant women, mothers  families, influential community members. |
| Salimi et al., 2012, [28] | Birth-related infection control | 3 | Cluster randomised control trial (1), randomised Control Trial (1), longitudinal quasi-experimental (1). | Nepal (1), Phillipines (1), Vietnam (1). | Women who had given birth, heterosexual male clients of commercial sex workers, high risk male heterosexual population, community leaders. |

*Criteria for inclusion; primary studies should include adult participants in CE interventions, include communicable disease control outcomes and include interventions from low or lower-middle income countries.
